# Black‐throated blue warblers (*Setophaga caerulescens*) exhibit diet flexibility and track seasonal changes in insect availability

**DOI:** 10.1002/ece3.70340

**Published:** 2024-09-20

**Authors:** Sara A. Kaiser, Lindsey E. Forg, Andrew N. Stillman, John F. Deitsch, T. Scott Sillett, Gemma V. Clucas

**Affiliations:** ^1^ Center for Biodiversity Sciences and Higher Education, Cornell Lab of Ornithology Cornell University Ithaca New York USA; ^2^ Department of Environment & Sustainability Cornell University Ithaca New York USA; ^3^ Center for Avian Population Studies, Cornell Lab of Ornithology Cornell University Ithaca New York USA; ^4^ Cornell Atkinson Center for Sustainability Cornell University Ithaca New York USA; ^5^ Department of Entomology Cornell University Ithaca New York USA; ^6^ Department of Biological Sciences Cornell University Ithaca New York USA; ^7^ Migratory Bird Center Smithsonian's National Zoo and Conservation Biology Institute Washington DC USA

**Keywords:** black‐throated blue warbler, climate change, diet flexibility, fecal DNA metabarcoding, phenology

## Abstract

Changes in leaf phenology from warming spring and autumn temperatures have lengthened the temperate zone growing “green” season and breeding window for migratory birds in North America. However, the fitness benefits of an extended breeding season will depend, in part, on whether species have sufficient dietary flexibility to accommodate seasonal changes in prey availability. We used fecal DNA metabarcoding to test the hypothesis that seasonal changes in the diets of the insectivorous, migratory black‐throated blue warbler (*Setophaga caerulescens*) track changes in the availability of arthropod prey at the Hubbard Brook Experimental Forest, New Hampshire, USA. We examined changes across the breeding season and along an elevation gradient encompassing a 2‐week difference in green season length. From 98 fecal samples, we identified 395 taxa from 17 arthropod orders; 242 were identified to species, with *Cecrita guttivitta* (saddled prominent moth), *Theridion frondeum* (eastern long‐legged cobweaver), and *Philodromus rufus* (white‐striped running crab spider) occurring at the highest frequency. We found significant differences in diet composition between survey periods and weak differences among elevation zones. Variance in diet composition was highest late in the season, and diet richness and diversity were highest early in the season. Diet composition was associated with changes in prey availability surveyed over the green season. However, several taxa occurred in diets more or less than expected relative to their frequency of occurrence from survey data, suggesting that prey selection or avoidance sometimes accompanies opportunistic foraging. This study demonstrates that black‐throated blue warblers exhibit diet flexibility and track seasonal changes in prey availability, which has implications for migratory bird responses to climate‐induced changes in insect communities with longer green seasons.

## Graphical abstract

Changes in seasonality have extended the breeding window for migratory birds in North America with earlier springs and later autumns that affect insect availability. DNA metabarcoding and insect surveys revealed that migratory, insectivorous black‐throated blue warblers exhibit diet flexibility in response to seasonal pulses in arthropod prey availability. They also express prey preferences, especially earlier in the season when birds are vulnerable to changes in insect availability.

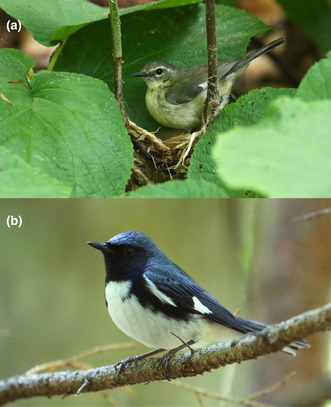

## 1 INTRODUCTION

The potential breeding window for migratory bird populations has extended in North America, with earlier leaf emergence and delayed leaf senescence due to climate change, which has lengthened the growing or “green” season in north temperate latitudes (Buitenwerf et al., 2015; Calinger & Curtis, 2023; Richardson et al., 2006; Zhu et al., 2012). Deciduous tree leaf phenology is mainly governed by temperature; leaf emergence is dictated by warming spring temperatures while cooling autumn temperatures activate the onset of leaf senescence (Richardson et al., 2006). Spring leaf phenology affects early seasonal events in the annual cycle of migratory birds, including the timing of spring arrival on the breeding grounds and the timing of breeding (Gordo, 2007; Vega et al., 2021). These behaviors have become progressively earlier over the last 50 years (Knudsen et al., 2011). Early breeding birds may have a fitness advantage over later breeding birds by providing more time to re‐nest following nest failure or raising additional broods after successfully rearing a first brood (Halupka et al., 2021; Townsend et al., 2013). However, this potential fitness benefit of an extended breeding season depends on whether late‐season food supplies are sufficient to support rearing additional broods (Husby et al., 2009; Nagy & Holmes, 2005a) and if species exhibit the flexibility in their diets to respond positively to potential changes in late season prey availability (Fufachev et al., 2019; Lowry et al., 2022; Mallord et al., 2017; Pilgrim et al., 2023).

Insectivorous birds are sensitive to the effects of changing seasonality on insect availability (Visser et al., 2006). Changes in leaf phenology from warming spring and autumn temperatures are associated with the earlier emergence and spring migration of insects, additional insect generations late in the growing season, and delayed autumn migration and insect diapause (Gallinat et al., 2015). These changes in early‐ and late‐season insect availability suggest that insectivorous birds might be able to take advantage of the fitness gains from multiple brooding and extend their breeding season (Halupka et al., 2021; Halupka & Halupka, 2017). Understanding how the diet composition and diet diversity of migratory birds vary over the green season will generate the mechanistic information necessary to better predict their responses to longer green seasons and associated changes in prey availability.

Advances in fecal DNA metabarcoding have revitalized studies of avian diets and trophic dynamics that could be affected by changing seasonality (Bumelis et al., 2022; Fayet et al., 2021; Hoenig et al., 2022; Spence et al., 2022; Stillman et al., 2022). DNA metabarcoding techniques use genetic markers to characterize the species composition and diversity of prey from a mixture of fragmented DNA found in fecal samples (Silva et al., 2019). These methods can have high taxonomic detectability and specificity relative to other methods for studying avian diets (Hoenig et al., 2022). They may enable the identification of rare prey species and soft‐bodied prey items, such as caterpillars, which are often overlooked using traditional methods that identify prey directly from foraging observations, feces, or stomach contents (Hoenig et al., 2022; Stillman et al., 2022). DNA metabarcoding is also a much less invasive technique than other methods, such as regurgitation studies, which can harm birds (Carlisle & Holberton, 2006). DNA‐based methods have important limitations, including a limited ability to quantify prey species abundance or biomass (Deagle et al., 2019) and to differentiate between larval and adult life stages of insect prey (Hoenig et al., 2022). Nonetheless, DNA metabarcoding can be used to characterize fine‐scale differences in the composition and diversity of diets, making such studies well‐suited for investigating dietary variation between early‐ and late‐breeding birds.

Here, we used fecal DNA metabarcoding to characterize within‐season spatial and temporal variation in the diet of the insectivorous, migratory black‐throated blue warbler (*Setophaga caerulescens*) during the green season at the Hubbard Brook Experimental Forest (Hubbard Brook), New Hampshire, USA. Since the 1980s, when research on this population began, leaf emergence dates have advanced 2.1 days per decade (8.4 days total) in response to increasing spring temperatures (Campbell et al., 2021; Richardson et al., 2006; Rustad et al., 2012), and autumn leaf senescence has occurred 2.8 days later per decade (11.2 days total) (Melaas et al., 2016), resulting in a 19.2 days extension of the green season. Black‐throated blue warblers have exhibited flexibility in their timing of arrival (advanced 0.16 ± 0.08 (SE) days per day of change in spring budburst) and breeding (advanced clutch initiation 0.56 ± 0.08 (SE) days per day of change in full spring leaf out) in response to earlier spring leaf emergence (Lany et al., 2016; Townsend et al., 2013) and have shown an increased propensity to double brood later in the breeding season (Germain et al., 2021), suggesting an extension of their breeding window both early and late in the season. However, the availability of prey can limit breeding season length by affecting the probability that females will initiate second broods (Kaiser et al., 2015; Nagy & Holmes, 2005a; Sillett, 2000). At Hubbard Brook, the seasonal pattern of arthropod availability for breeding birds (i.e., caterpillars and flying insects) is highly variable throughout the breeding season due to the changing availability of prey that have diverse life histories and larval feeding times (Lany et al., 2016; Stange et al., 2011). Thus, to understand the capacity of black‐throated blue warblers to respond to potential changes in prey availability with a lengthening green season, we examined variability in their diets corresponding to spatial and temporal flushes in arthropod availability. Moreover, the age distribution of black‐throated blue warblers is associated with variation in habitat quality (i.e., shrub density and insect availability) along an elevation gradient encompassing a 2‐week difference in green season length. Older (≥2 years of age) black‐throated blue warblers generally occupy higher quality habitats at higher elevations where the green season length is longer, and younger individuals settle in less suitable habitats. This results in differential reproductive output, mainly through double brooding of older individuals (Holmes et al., 1996; Kaiser et al., 2015). These habitat occupancy patterns suggest that diet composition may differ between older and younger breeding birds, especially early and late in the season.

Our objectives were to (1) provide a baseline description of the composition, diversity, and intra‐seasonal variation of black‐throated blue warbler diets at Hubbard Brook; (2) compare diet composition and diversity among age groups; and (3) compare diets with insect availability based on results from two separate field methods to survey insects. We used the combination of diet and survey data to test the hypothesis that warbler diets respond to changes in the underlying availability of arthropod prey. If warbler diets track prey availability, we predicted that the arthropod taxa detected in diets would occur in proportion to their availability in three separate survey periods spanning the breeding and post‐fledging periods. Alternatively, if warblers show strong selection for certain prey relative to availability, we predicted that favored prey items would occur in warbler diets in greater proportion than expected based on their availability.

## 2 MATERIALS AND METHODS

### 2.1 Study population

**FIGURE 1:**
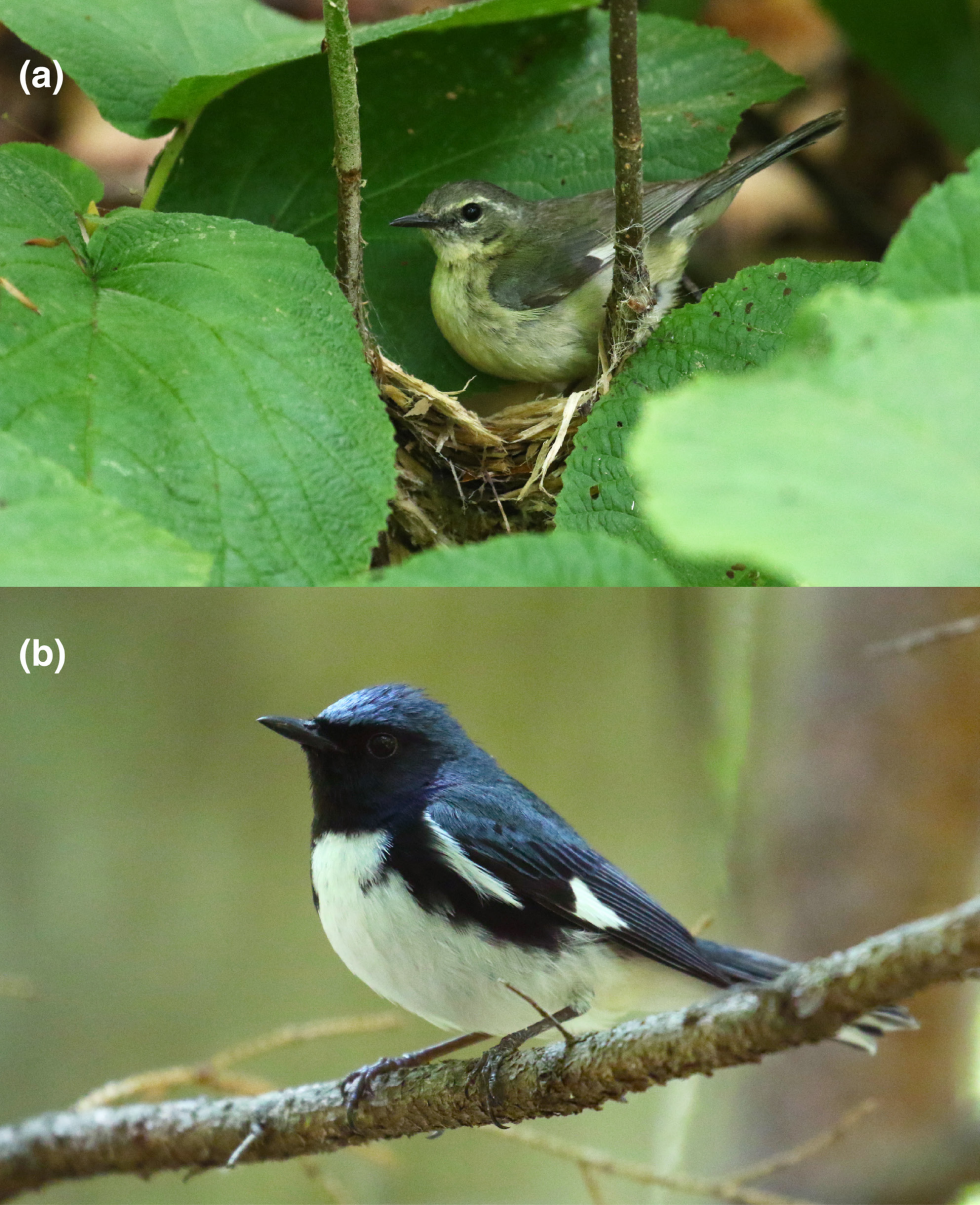
Black‐throated blue warbler (*Setophaga caerulescens*) (a) female building her nest and (b) male perched in the understory of the Hubbard Brook Experimental Forest, New Hampshire, USA. Photos: John F. Deitsch.

We studied the diet of the insectivorous, migratory black‐throated blue warbler (Figure 1) as part of a larger demographic study of this species at the 3160 ha Hubbard Brook Experimental Forest (Hubbard Brook), New Hampshire, USA (43°56′N, 71°45′W). The study area is a northern hardwood forest spanning a 600‐m elevation gradient with an overstory dominated by sugar maple (*Acer saccharum*), American beech (*Fagus grandifolia*), and yellow birch (*Betula alleghaniensis*), with red spruce (*Picea rubens*), balsam fir (*Abies balsamea*), and white birch (*B. papyrifera*) increasing in abundance at higher elevations (Schwarz et al., 2003; van Doorn et al., 2011). The shrub layer is dominated by hobblebush (*Viburnum lantanoides*), the preferred nest substrate for the warblers (Holmes et al., 2020), along with saplings of striped maple (*Acer pensylvanicum*) and the major canopy species (Schwarz et al., 2003; van Doorn et al., 2011). Black‐throated blue warblers primarily forage by gleaning prey from foliage in the shrub and lower forest canopy (Robinson & Holmes, 1982). Foraging observations and earlier studies of their stomach contents at Hubbard Brook indicate they consume Lepidoptera larvae and adults, adult Diptera, Coleoptera, spiders, and other arthropods, including Homoptera and Hymenoptera, and small snails (Holmes et al., 1986; Robinson & Holmes, 1982).

The breeding ecology of the black‐throated blue warbler has been studied extensively at Hubbard Brook (Holmes, 2007, 2011; Holmes et al., 2020; Holmes & Likens, 2016). Males and females arrive in early May, establish territories (1–4 ha), pair, and initiate breeding in mid‐ to late May, depending on spring leaf expansion (Holmes et al., 2020; Lany et al., 2016). Females build open cup nests <1 m from the ground in the shrub layer, lay one egg per day (mean clutch size = 3.6, range = 2–5 eggs), and incubate for 12 days. Both females and males will feed nestlings for 9 days until fledging and continue to feed fledglings for 3 weeks or until they become independent (Holmes et al., 2020). Small nestlings are most commonly fed small flying insects (Diptera and Hymenoptera), spiders, and small, smooth‐skinned caterpillars (Holmes et al., 2020). In contrast, larger nestlings are fed crane flies, moths, and small and large caterpillars (Holmes et al., 2020; Rodenhouse & Holmes, 1992). The diets of fledglings have not been characterized. At Hubbard Brook, one‐third of black‐throated blue warbler pairs attempt second broods (i.e., double brood) when food resources are abundant in the late season (i.e., late July) (Kaiser et al., 2015; Nagy & Holmes, 2005b; Townsend et al., 2013), with more birds double brooding at higher elevations where food is less limiting (Kaiser et al., 2015, 2017).

### 2.2 Sample collection

We collected data on a 330‐ha study plot at three elevation zones (low: 380–499 m [85 ha], mid: 500–599 m [120 ha], high: 600–740 m [125 ha]) during three 4‐week survey periods from May–Aug 2021 (early: 15 May–15 June, mid: 16 June–15 July, and late: 16 July–15 August) (Table S1). We mapped the birds' territories, captured and marked adults, collected fecal samples from adults, and found and monitored nesting attempts. The boundaries of male territories were mapped throughout the breeding season relative to a 50 × 50 m virtual grid by recording the locations of singing males and agonistic encounters between neighboring males on a handheld GPS unit (Garmin, Olathe, KS). All individuals were captured within their territories between 0600 and 1400 and marked with a unique combination of three colored leg bands and one U.S. Geological Survey leg band. We found nests by following females during nest building, following adults carrying food, and searching vegetation. We monitored nests throughout all nest stages every other day, with daily checks near clutch completion and anticipated hatch and fledge dates. Males and females were aged as hatch year (HY), second year (SY), or after second year (ASY) breeders based on plumage characteristics (i.e., replaced or retained primary coverts, remiges, and rectrices) (Holmes et al., 2020). Males were lured into 6‐m mist nets with song playback and a male decoy. Females were captured by flushing them off their nests into a 6‐m net placed in front of their nest. At capture, adults were placed in a fresh paper bag with an index card at the bottom for a maximum of 10 min or until defecation. Fecal samples were collected off the paper bag or index card into sterile 2 mL tubes containing 1 mL DNA/RNA Shield (Zymo Research, Irvine, CA), stored at room temperature until the end of the field season, and transferred to a −20°C freezer in an ancient DNA lab until DNA extraction in September–October 2021. We captured 44 birds that did not defecate after being held for 10 min that we released without obtaining a sample. All banders sterilized their hands with alcohol wipes after handling each bird to minimize the risk of cross‐contamination. We collected field blanks at each elevation and during each survey period to monitor for background contamination throughout the study. Field blanks were collected by scraping a clean paper bag or index card with a sterile 2 mL tube containing 1 mL DNA/RNA Shield, mimicking sample collection. Field methods at Hubbard Brook were approved by the Institutional Animal Care and Use Committee (IACUC) at Cornell University (Protocol 2009‐0133) and conducted under scientific permits issued by the USGS Bird Banding Laboratory (Permit 24243) and New Hampshire Fish and Game Department (Permit RSA 214:29).

### 2.3 Insect availability

We examined spatial and temporal variation in insect availability across the same three 4‐week survey periods from 15 May to 15 August and three elevation zones. We conducted visual caterpillar surveys in the shrub layer along 12 line transects (4 transects at each of the three elevation zones) during six 2‐week survey periods (15–31 May, 1–15 June, 16–30 June, 1–15 July, 16–31 July, and 1–15 August) (Holmes et al., 1979; Holmes & Schultz, 1988). We searched for caterpillars on 100 leaves each of striped maple, American beech, and hobblebush at 10 points along each line transect (1000 leaves/transect for each tree species). Caterpillars were identified to family, counted, and measured to the nearest mm. We converted caterpillar measurements to wet biomass (mg) using length‐mass regressions (Rogers et al., 1977). For three focal Lepidoptera families (Geometridae [geometer moths], Noctuidae [owlet moths, armyworms, cutworms], and Notodontidae [prominent moths]), we summed caterpillar biomass across the four transects (caterpillar biomass/12,000 leaves) sampled within a 2‐week survey period at each elevation zone. To facilitate comparisons between caterpillar survey results and fecal samples, we grouped 2‐week caterpillar survey data into three 4‐week survey periods (early, mid, and late) matching the three temporal windows of fecal sampling. We calculated the proportional biomass of each Lepidoptera family sampled during the early, mid, and late survey periods.

We collected two 24‐h samples of flying insects each week from two Malaise traps at each of three elevation zones over the 12‐week season (total = 72 Malaise samples). Malaise samples were sorted, and taxa were identified to order and family, when possible (Rodenhouse & Holmes, 1992). We counted the number of individuals >4 mm in length in each taxonomic group sampled in Malaise traps and summed across eight samples collected within each 4‐week survey period (early, mid, and late; as above) at each elevation zone. Although Malaise traps do collect spiders (Araneae) and beetles (Coleoptera), they are designed to sample flying insects. We removed Araneae and Coleoptera from the dataset because of potential bias in sampling these taxa using only Malaise traps (Montgomery et al., 2021; Oxbrough et al., 2010). Thus, the frequency of occurrence of taxa included in the dataset reflects the availability of these taxa to foraging warblers with high confidence. The resulting dataset included 13 taxonomic groups: Hemiptera Pentatomidae (stink bugs and shield bugs), Hemiptera Other (true bugs such as planthoppers and assassin bugs), Homoptera Leafhopper (leafhoppers), Homoptera Other, Plecoptera (stoneflies), Trichoptera (caddisflies), Lepidoptera (butterflies and moths), Diptera Tipulidae (crane flies), Diptera Rhagionidae (snipe flies), Diptera Other (horse‐flies, hoverflies, and others), Hymenoptera Ichneumonidae (parasitoid wasps), Hymenoptera Other (sawflies, wasps, bees, and ants), and Mecoptera Panorpidae (scorpionflies). We calculated the frequency of occurrence of each taxonomic group sampled during the early, mid, and late survey periods.

### 2.4 Molecular lab work

We used DNA metabarcoding to characterize diet composition with high taxonomic specificity (Garfinkel et al., 2020). We randomly placed samples into groups spanning elevation zones and survey periods for DNA extractions to avoid batch effects. Samples were homogenized through bead beating on a benchtop vortex for 20 min before extraction. We extracted fecal DNA in a clean lab with ultraviolet decontamination using a Quick‐DNA Fecal/Soil Microbe MiniPrep Kit following the manufacturer's protocol (Zymo Research, Irvine, CA), with an elution volume of 75 μL. For each group of extractions, we included one negative extraction control and one positive insect control (i.e., mock prey community DNA). The positive insect control was created by selecting five insect families collected from Malaise traps that represent known prey items of black‐throated blue warblers at Hubbard Brook (Cantharidae, Cerambicidae, Curculionidae, Ichneumonidae, Rhagionidae). We extracted DNA from the legs and heads of insects using the DNeasy Blood & Tissue Kit (Qiagen, Hilden, Germany), with an elution volume of 100 μL.

We used a two‐step PCR protocol with the ANML primers (Jusino et al., 2019) to amplify a 200 base pair sequence of the mitochondrial gene, cytochrome oxidase C subunit 1 (COI), a mitochondrial marker frequently used in avian diet analyses to identify dietary components (Hoenig et al., 2022). The ANML primer pair is more effective than other primers, such as ZBJ, because it has better taxonomic coverage (Forsman et al., 2022; Jusino et al., 2019). Forward and reverse ANML primers were modified for metabarcoding by adding TruSeq tails for use in the second PCR. We ran PCRs on 96‐well plates, including fecal samples, field blanks, negative extraction controls, one positive insect control, and two no‐template PCR negative controls. PCR reactions were performed in 25 μL reactions with 12.5 μL AmpliTaq Gold 360 Master Mix (Applied Biosystems, Waltham, MA), 1 μL forward primer (10 μM), 1 μL reverse primer (10 μM), and 10.5 μL template (or 10.5 μL water for the no‐template negative control). The positive insect control contained 9.5 μL water and 1 μL mock prey community DNA, which was made by mixing 10 μL of each of the 5 insect families chosen. We ran PCRs in duplicate to minimize errors from false negatives or poor amplification (Taberlet, 1996). We used the following thermocycling conditions: an initial denaturation step at 95°C for 10 min followed by 35 cycles of 95°C for 30 s, 50°C for 30 s, 72°C for 30 s, and a final extension of 72°C for 7 min. PCR products were visualized using gel electrophoresis, and duplicate PCR runs were combined before being diluted based on band brightness. Samples with bright bands were diluted in a 1:10 ratio with molecular grade water, samples with intermediately bright bands were diluted 1:5, and samples with faint or no bands were diluted in a 1:3 ratio. Before sequencing at the Hubbard Center for Genome Studies (Durham, New Hampshire), a second‐step PCR was performed to add unique dual indexes to each sample. Sequencing was performed using ~5% of a NovaSeq lane.

### 2.5 Bioinformatics and data processing

We processed the sequences using Qiime2 v2021.4 (Bolyen et al., 2019). We first checked the read quality and number of reads per plate before trimming primers from the 3′ and the 5′ ends of the reads using the *cutadapt* plugin (Martin, 2011). Next, we filtered out low‐quality reads and denoised the reads using the *DADA2* plugin (Callahan et al., 2016), truncating the length of sequences to 130 bp and specifying a minimum overlap of 50 bp between the forward and reverse reads. After denoising, we merged the sequence reads from each plate of samples and assigned taxonomy to the sequences using a pre‐trained COI classifier. This naïve Bayes classifier was trained on COI sequences downloaded and curated from the Barcode of Life Database (BOLD) using the *RESCRIPt* plugin (Robeson et al., 2021) and trimmed to the ANML region by O'Rourke et al. (2020). Following the taxonomy assignment, we removed non‐arthropod reads (0.7% of reads) and calculated alpha‐rarefaction curves to determine rarefaction depth. The non‐arthropod reads were identified as either unassigned to any taxon, undetermined Animalia, Fungi, Nematoda, Tardigrada, Heterokontophyta (Protozoa), Mollusca, or Rotifera, and we omitted them because the ANML primers are primarily suitable for detecting arthropod COI sequences. We used the mock community to confirm the correct taxonomic assignment of dominant prey families documented at Hubbard Brook. Assigned prey species were compared to the species list of arthropods known to occur at Hubbard Brook or in the Northeast region. For any prey species that had not been documented in this region, we compared prey sequences to the National Center for Biotechnology Information's Genbank (https://www.ncbi.nlm.nih.gov/genbank/) using BLASTn (Basic Local Alignment Search Tool) to determine percentage identity and query coverage. If the percentage identity was below 98%, we adjusted the taxonomic classification to a higher rank (e.g., from species to genus).

### 2.6 Data analysis

#### 2.6.1 Diet composition

We characterized black‐throated blue warbler diets using the presence–absence data to reduce biases associated with sequence read abundances (Deagle et al., 2019; Jusino et al., 2019). To examine the factors affecting diet composition, we performed PERMANOVA at the taxonomic level of prey families using the *adonis2* function from the *vegan* package in R version 4.4.0 (Oksanen et al., 2019; R Core Team, 2024) The right‐hand side of the formula included independent variables for survey period, elevation, age group, and the interaction between survey period and elevation. We controlled for potential differences between sexes using a blocked design where permutations were constrained within each sex. We investigated differences in multivariate dispersion between groupings of samples using the *betadisper* function in *vegan* (Anderson, 2006), followed by Tukey tests to conduct pairwise comparisons of mean dispersions. This approach tests for heterogenous variances between groups using the average distance of group members to a group‐specific centroid in multidimensional space. Next, we visualized dietary niche space using non‐metric multidimensional scaling (NMDS). All comparisons used the modified Raup‐Crick dissimilarity index for the presence–absence data (Chase et al., 2011). We separately summarized the frequency of occurrence for each prey family for the early, mid, and late survey periods.

#### 2.6.2 Diet richness and diversity

We compared diet richness and diversity between survey periods using rarefaction curves to interpolate to the lowest common sample size (*n* = 24; R package iNEXT; Hsieh et al., 2020) All comparisons used species‐level prey taxonomies to generate estimates of diet richness, Shannon diversity (exponentiated Shannon entropy), and Simpson diversity (inverse Simpson concentration). We used 95% confidence bands around estimates to interpret differences between groups (Chao et al., 2014). In addition, we tested for differences in per‐sample prey species richness for the early, mid, and late survey periods using a Kruskal–Wallis test.

#### 2.6.3 Prey selection

In our study, warbler fecal sampling accompanied two types of surveys to sample arthropod communities throughout the green season and across elevation zones: visual caterpillar surveys and Malaise traps. We used these survey data to test the hypothesis that diets respond to changes in the underlying availability of arthropod prey and the alternative hypothesis that warblers show prey selectivity. We compared diet data to prey availability using three separate approaches.

For the first approach, we used the caterpillar survey results to calculate the proportional biomass of three Lepidopteran families in each survey period (proportional biomass = family‐specific biomass/total biomass * 100). Plotting the changes in frequency of occurrence in samples and proportional biomass between survey periods highlights potential temporal patterns in prey selection or avoidance and provides a simple visual comparison between diet and availability.

Second, we used a linear modeling approach to compare the temporal patterns of Malaise trap results to fecal samples and test the hypothesis that, on average, diet tracked the availability of flying insects across survey periods. We organized taxa identified in the fecal samples into the same 13 taxonomic groups sampled by Malaise traps and calculated a taxon‐specific frequency of occurrence for each survey period. Next, we calculated the Malaise capture frequency for each taxon by dividing the number of captures by the total captures. Here, values represent the percent of the total captures in each survey period. Using each taxon and survey period combination as a sampling unit (*n* = 42), we modeled the linear relationship between fecal frequency of occurrence in samples and Malaise capture frequency. A positive slope estimate indicates that prey in the diet generally track availability, and residual outliers show evidence for selection or avoidance of specific taxa.

For the third approach, we characterized the taxon‐specific patterns of prey selection and avoidance through the three survey periods using the R package econullnetr (R Core Team, 2024; Vaughan et al., 2018). This method uses a null modeling approach to create taxon‐specific hypothesis tests based on the observed frequency of occurrence of prey items in diet samples compared to availability from field surveys. The null model uses data on prey availability to simulate the potential frequency of occurrence in the absence of prey selection and compares these simulations to the observed data. Taxonomic groups that occur in the diet more frequently than expected suggest prey selection and groups that occur less frequently than expected suggest prey avoidance. We developed null models using data from caterpillar surveys (3 focal Lepidoptera taxa; Table S2) and Malaise traps separately, and we ran each null model for 999 iterations. Starting with the 13 taxonomic groups identified from Malaise surveys, we additionally excluded four taxa with fewer than 15 total Malaise trap captures (Hemiptera Pentatomidae, Hemiptera Other, Homoptera Leafhopper, and Homoptera Other) to avoid basing statistical inference on taxa with low sample sizes (Table S3).

## 3 RESULTS

From May to August 2021, we collected fecal samples from 99 black‐throated blue warblers and successfully recovered arthropod DNA from 98 of the samples (Table S1). Illumina sequencing generated 34 million COI sequences with 25 million reads passing quality filters. We rarefied samples and removed 11 samples that lacked sufficient depth to capture all prey taxa (<23,000 reads), resulting in 87 samples in the diet dataset. Six of our extraction blanks and field negative controls (out of 20) yielded sequences with an average sequencing depth of 0.04% compared to our diet samples (controls yielded an average of 1013 reads compared to an average of 220,000 reads from diet samples). This suggests that contamination from the field and lab was not pervasive in our dataset and only very minor when it did occur, resulting in all controls being dropped when we set our rarefaction threshold of 23,000 reads. We identified 395 distinct arthropod taxa belonging to 17 orders representing 121 families, from which nine prey were identified to genus, and 242 prey were identified to species. The orders with the highest frequency of occurrence (species that occur in ≥20% of samples) were Lepidoptera (100%), Araneae (98.9%), Diptera (94.3%), Hemiptera (62.1%), Hymenoptera (44.8%), Psocodea (34.5%), and Coleoptera (28.7%) (Table S4). Most taxa were rare; 192 (48.6%) were detected in only one sample. All orders in the mock community were correctly identified.

### 3.1 Diet composition

**FIGURE 2:**
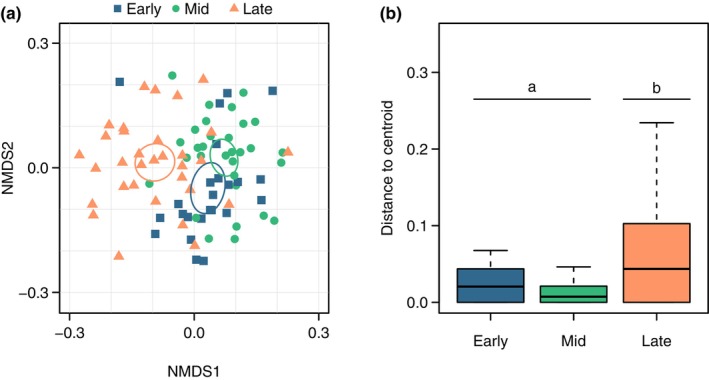
(a) Non‐metric multidimensional scaling (NMDS) plot of diet composition shows a limited overlap between the diets of black‐throated blue warblers during the late survey period relative to the early‐ and mid‐survey periods at the Hubbard Brook Experimental Forest, New Hampshire, USA. Points on the NMDS plot represent the diet composition of individual samples based on prey species displayed with 95% confidence ellipses. (b) Distances to the ordination centroid show that multivariate dispersion was greater in the late survey samples relative to early‐ and mid‐survey samples. Boxes represent 25–75% interquartile ranges (IQR), lines represent medians, and whiskers represent data range excluding outliers (>1.5 times IQR).

The family‐level composition of black‐throated blue warbler diets differed significantly between survey periods (Table 1). NMDS ordination generated a 3‐dimensional solution (stress = 0.23) that highlighted differences in diet composition in the late survey period compared to the early and mid‐survey periods (Figure 2). Diet composition also showed limited overlap between age groups (HY relative to SY and ASY; Table 1: PERMANOVA, Figure S1a), although the sample size of HY birds was small (*n* = 5), and all samples were collected in the late survey period. Diet composition showed some overlap between elevation zones (Table 1: PERMANOVA, Figure S1b), but we detected no interaction effect between the survey period and elevation zone (Table 1). Variation within diets differed based on the survey period but not the elevation zone or age group (Table 1: Multivariate dispersion). The variability in diet increased as the green season progressed, and pairwise comparisons between survey periods demonstrated that mean multivariate dispersion was greater in the late survey period compared to the early (*p* = .042) and mid (*p* < .001) survey periods (Table 1; Table S5).

**TABLE 1:** PERMANOVA results indicate significant dissimilarity in black‐throated blue warbler diets between survey periods and age groups.

Parameter	PERMANOVA			Multivariate dispersion	
	Pseudo‐*F*	*R* ^2^	*p*‐value	*F*	*p*‐value
Survey period	10.6	.18	.036	7.6	<.001
Elevation	8.4	.14	.074	0.1	.903
Age group	10.4	.17	.043	1.0	.386
Survey period * elevation	−4.0	−.14	.988		

*Note*: All variables contained three levels (i.e., balanced design) and tests were conducted at the level of prey species.

**FIGURE 3:**
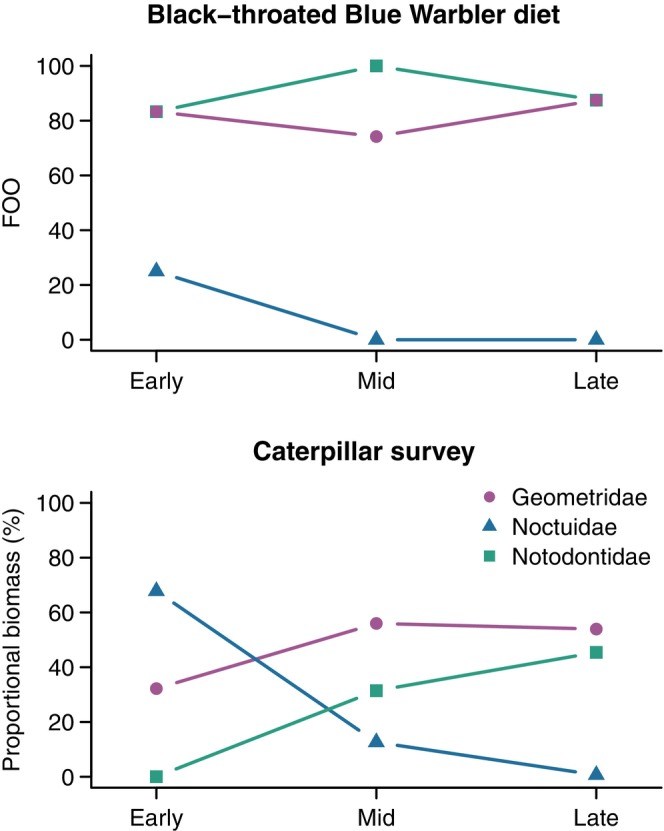
Within‐season patterns in prey frequency of occurrence in black‐throated blue warbler diets (top) compared to prey availability (proportional biomass) for three focal Lepidoptera families surveyed on transects (bottom) at the Hubbard Brook Experimental Forest, New Hampshire, USA.

Black‐throated blue warblers primarily selected arthropod prey from the Order Lepidoptera, present in all fecal samples collected over the green season (Table 2). Within Lepidoptera, Notodontidae (prominent moths; 79% of samples) and Geometridae (geometer moths; 71%) were the most frequently detected prey families across survey periods. Tortricidae (Tortricid leafroller moths) were present in moderate frequency in samples from the early (62.5%) and mid (67.7%) survey periods but were substantially less frequent in samples from late in the season (18.8%). In contrast, some Lepidopteran families (e.g., Noctuidae) that occurred in low frequency in the samples were highest in the early survey period (<20%) but very low or not present in the mid and late survey periods (Figure 3, Table S6). The frequency of occurrence of Arachnids in samples was generally high in early and mid‐survey periods but low late in the season (Table 2). For example, Theridiiadae (cobweb spiders) were frequently present in samples from the early (91.7%) and mid (93.5%) survey periods but decreased late in the season (53.1%). Similar patterns were observed for Arachnids that were less frequent in samples (e.g., Araneidae [orbweavers] and Philodromidae [running crab spiders]; Table 2). Hemiptera, Diptera, and Hymenoptera orders had the lowest frequency of occurrence, with a single family of Hymenoptera (Ichneumonidae) detected more frequently in samples from the late survey period. The complete list of detected arthropod prey families is given in Table S6.

**TABLE 2:** Frequency of occurrence (%) of the top 17 prey families (families that occur in ≥20% of samples) identified in the diets of black‐throated blue warblers in each survey period (early, mid, and late) at the Hubbard Brook Experimental Forest, New Hampshire, USA.

Class	Order	Family	Common Name	% Frequency of occurrence			
				Total	Early	Mid	Late
				*n* = 87	*n* = 24	*n* = 31	*n* = 32
Insecta	Lepidoptera	Notodontidae	Prominent moths	79.0	83.3	100	87.5
Insecta	Lepidoptera	Geometridae	Geometer moths	71.0	83.3	74.2	87.5
Arachnida	Araneae	Theridiidae	Cobweb spiders	68.0	91.7	93.5	53.1
Arachnida	Araneae	Araneidae	Orbweavers	53.0	79.2	64.5	43.8
Arachnida	Araneae	Philodromidae	Running crab spiders	53.0	83.3	71.0	34.4
Insecta	Lepidoptera	Tortricidae	Tortricid leafroller moths	42.0	62.5	67.7	18.8
Arachnida	Araneae	Linyphiidae	Sheetweb dwarf weavers	41.0	54.2	32.3	56.2
Insecta	Hemiptera	Miridae	Plant bugs	36.0	25	74.2	21.9
Insecta	Diptera	Rhagionidae	Snipe flies	33.0	62.5	22.6	34.4
Arachnida	Araneae	Dictynidae	Meshweavers	32.0	50.0	41.9	21.9
Insecta	Hymenoptera	Ichneumonidae	Ichneumonid wasps	30.0	29.2	29.0	43.8
Insecta	Diptera	Cecidomyiidae	Gall and forest midges	29.0	37.5	32.3	31.2
Insecta	Diptera	Tachinidae	Bristle flies	24.0	16.7	29.0	34.4
Insecta	Diptera	Mycetophilidae	Fungus gnats	22.0	8.3	16.1	46.9
Arachnida	Araneae	Tetragnathidae	Long‐jawed orbweavers	22.0	50.0	22.6	9.4
Insecta	Hemiptera	Cicadellidae	Typical leafhoppers	20.0	20.8	22.6	25.0
Insecta	Lepidoptera	Gelechiidae	Twirler moths	20.0	33.3	25.8	12.5

*Note*: Taxonomy was assigned from the Barcode of Life Database (BOLD). Each row represents a different family. Total frequency is the percentage of the total number of samples across survey periods that included a given family. The full list of detected prey families is given in Table S6.

The frequency of occurrence of dominant prey species in samples differed across the three survey periods (Table 3), contributing to the observed differences in dietary composition over the green season. The prey species that occurred in the highest frequency of samples across all survey periods were *Cecrita guttivitta* (saddled prominent moth, 79% of samples), followed by two species of spiders, *Theridion frondeum* (eastern long‐legged cobweaver; 53%) and *Philodromus rufus* (white‐striped running crab spider; 47%), that decreased in samples from early to late season (Table 3). The complete list of detected arthropod prey species is given in Table S7.

**TABLE 3:** Frequency of occurrence (%) of the top 19 prey species (species that occur in ≥20% of samples) identified in the diets of black‐throated blue warblers in each survey period (early, mid, and late) at the Hubbard Brook Experimental Forest, New Hampshire, USA.

Phylum	Class	Order	Family	Species	Common name	% Frequency of occurrence			
						Total	Early	Mid	Late
						*n* = 87	*n* = 24	*n* = 31	*n* = 32
Arthropoda	Insecta	Lepidoptera	Notodontidae	*Cecrita guttivitta*	Saddled Prominent Moth	79.0	83.3	100	87.5
Arthropoda	Arachnida	Araneae	Theridiidae	*Theridion frondeum*	Eastern Long‐legged Cobweaver	53.0	70.8	93.5	21.9
Arthropoda	Arachnida	Araneae	Philodromidae	*Philodromus rufus*	Running Crab Spider	47.0	75.0	64.5	28.1
Arthropoda	Insecta				Insect	43.0	62.5	41.9	46.9
Arthropoda	Insecta	Lepidoptera			Butterfly/Moth	33.0	54.2	51.6	12.5
Arthropoda	Arachnida	Araneae	Araneidae	*Cyclosa conica*	Conical Trashline Orbweaver	33.0	70.8	32.3	18.8
Arthropoda	Arachnida	Araneae	Dictynidae	*Emblyna maxima*	Cribellate Araneomorph Spider	30.0	45.8	41.9	18.8
Arthropoda	Insecta	Lepidoptera	Geometridae	*Orthofidonia exornata*	Geometrid Moth	28.0	4.2	25.8	59.4
Arthropoda					Arthropod	28.0	37.5	6.5	53.1
Arthropoda	Insecta	Lepidoptera	Tortricidae	*Pandemis lamprosona*	Woodgrain Leafroller Moth	27.0	33.3	51.6	9.4
Arthropoda	Insecta	Lepidoptera	Geometridae		Geometrid Moth	26.0	4.2	25.8	53.1
Arthropoda	Insecta	Diptera	Cecidomyiidae		Gall Gnat	25.0	37.5	29	21.9
Arthropoda	Arachnida	Araneae	Tetragnathidae	*Tetragnatha shoshone*	Spider	22.0	50.0	22.6	9.4
Arthropoda	Insecta	Hemiptera	Miridae	*Deraecoris grandis*	Plant Bug	20.0	16.7	48.4	3.1
Arthropoda	Arachnida				Spider	20.0	12.5	12.9	40.6
Arthropoda	Arachnida	Araneae	Linyphiidae	*Helophora insignis*	Sheetweb Spider	20.0	4.2	12.9	46.9
Arthropoda	Arachnida	Araneae	Philodromidae	*Philodromus praelustris*	Running Crab Spider	20.0	37.5	19.4	15.6
Arthropoda	Arachnida	Araneae	Araneidae	*Araneus saevus*	Fierce Orbweaver	20.0	33.3	9.7	28.1
Arthropoda	Arachnida	Araneae	Araneidae	*Araneus guttulatus*	Red‐backed Orbweaver	20.0	12.5	35.5	18.8

*Note*: Taxonomy was assigned from the Barcode of Life Database (BOLD). Each row represents a different species, identified to the highest possible taxonomic rank. Total frequency is the percentage of the total number of samples across survey periods that included a given species. The full list of detected prey species is given in Table S7.

Although the sample size of HY birds was small (*n* = 5) compared to SY (*n* = 49) and ASY (*n* = 31) birds, diet composition suggests significant differences between age groups (HY relative to SY and ASY; Table 1, Figure S1a). Because all samples from HY birds were collected in the late survey period, we conducted a *post‐hoc* PERMANOVA analysis with HY birds omitted to test whether our conclusions were robust to potential confounding between age and the survey period. The results were consistent when HY birds were removed from the analysis (Table S8). Notably, during the late survey period, two orders with a higher frequency of occurrence in HY birds relative to SY/ASY birds were Psocodea (lice) and Trombidiformes (mites) (Table S9).

### 3.2 Diet richness and diversity

Individual samples contained a median of four orders (range = 1–8, *n* = 17), 12 families (range = 4–26, *n* = 121), and 18 species (range = 5–40, *n* = 242). We found weak differences between survey periods in the number of prey species present in each sample (*χ* ^2^ = 5.25, df = 2, *p* = .072). Samples from the early survey period had the highest per‐sample richness on average (Figure S2). For all samples combined, rarefied prey richness estimates were substantially higher in the early survey period (203 species, *n* = 24) and lower in the mid (164 species, interpolated *n* = 24) and late (165 species, interpolated *n* = 24) survey periods (Table S10). Similarly, Shannon and Simpson's diversity of prey items was highest in the early survey period, but Simpson's diversity values showed considerable overlap in confidence intervals between survey periods (Table S10).

### 3.3 Prey selection

We sampled a total of 1567 caterpillars along line transects over the three survey periods from three focal Lepidopteran families (Geometridae = 1035, size range = 1–38 mm; Notodontidae = 466, size range = 1–36 mm; Noctuidae = 66, size range = 3–23 mm). The proportional biomass of Geometridae and Notodontidae caterpillars increased over the green season, yet the high occurrence of these taxa in warbler diets remained notably consistent (Figure 3). The occurrence of Noctuids in fecal samples was much lower than expected in the early survey period when availability was relatively high (Figure 3). In the early‐ and mid‐survey periods, warblers showed positive selection for Notodontidae and avoidance of Noctuidae, with no evidence for prey selection or avoidance during the late survey period (Figure 5).

**FIGURE 4:**
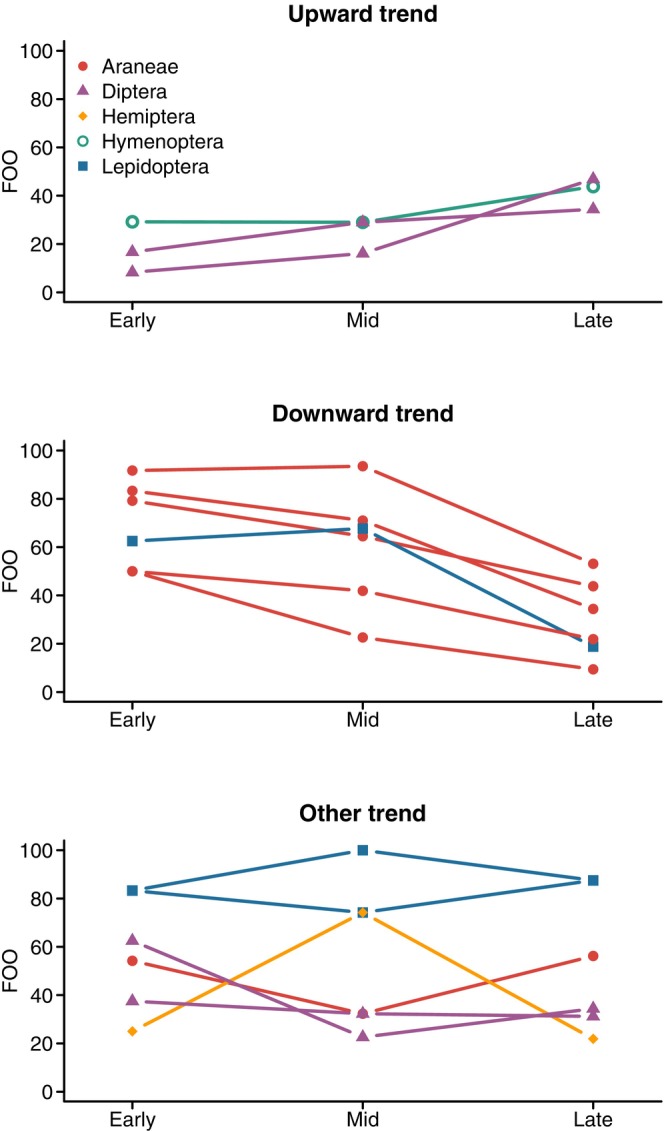
Frequency of occurrence for prey items in the diets of black‐throated blue warblers at the Hubbard Brook Experimental Forest, New Hampshire, USA. Lines are shown for the 15 families, colored by order, with the highest frequencies of occurrence in fecal samples. Panels highlight examples of upward, downward, or “other” temporal patterns across survey periods.

**FIGURE 5:**
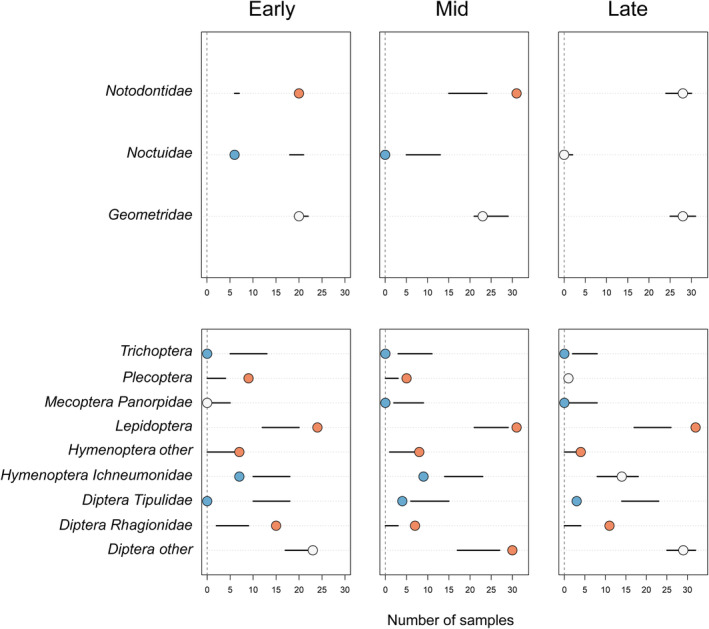
Dietary preferences of black‐throated blue warblers show changing patterns of prey selection through the early, mid, and late sampling periods compared to field data from caterpillar surveys and Malaise traps at the Hubbard Brook Experimental Forest, New Hampshire, USA. Horizontal axes show the number of fecal samples with each taxonomic prey group. Black lines indicate the 95% confidence interval predictions from the null model based on insect availability, and dots denote observed diet frequencies. Blue dots represent prey consumed less than expected, orange dots represent prey consumed more than expected, and white dots show prey consumed in proportion to availability.

We sampled a total of 2300 arthropods >4 mm in Malaise traps over the three survey periods from 13 focal taxa (Diptera Other = 916, Lepidoptera = 506, Diptera Tipulidae = 287, Hymenoptera Ichneumonidae = 287, Trichoptera = 100, Mecoptera Panorpidae = 48, Diptera Rhagionidae = 37, Hymenoptera Other = 29, Plecoptera = 15, Hemiptera Other = 4, Homoptera Leafhopper (leafhoppers) = 4, and Hemiptera Pentatomidae = 3). These taxa in warbler diets showed variable trends in their frequency of occurrence among survey periods (Figure 4). Among these taxa, the frequency of occurrence in warbler diets showed a strong, positive relationship with Malaise trap capture frequencies (Figure S3, slope = 0.24, 95% CI = 0.15, 0.32). Here, a positive slope supports a general pattern of diet composition tracking the availability of prey on average across taxa. However, null model comparisons showed taxon‐specific patterns of prey selection or avoidance (Figure 5), with no significant evidence that these patterns changed between survey periods. For example, warblers were selected for Diptera Rhagionidae and avoided Diptera Tipulidae in all three survey periods.

## 4 DISCUSSION

### 4.1 Summary

Black‐throated blue warblers exhibited seasonal flexibility in their diets with marked differences in arthropod prey consumed early and late in the green season. Diet composition differed significantly between survey periods, with greater variability late in the season and weak differences among elevation zones. Dietary richness and diversity decreased over the green season, with higher diversity early in the season. Significant differences in diet between juveniles and adults contributed to diet variation during the late season, although a small HY sample size prevented us from exploring this further. Furthermore, our results support both hypotheses; black‐throated blue warbler diets track seasonal changes in arthropod availability, but several taxa were consistently selected or avoided as prey across survey periods. However, Notodontid caterpillars were selected earlier in the season when their abundance was low, which has implications for possible changes in the availability of these insect prey with the extension of the breeding window from earlier springs and later autumns at Hubbard Brook (Campbell et al., 2021; Melaas et al., 2016; Richardson et al., 2006; Rustad et al., 2012). We acknowledge that our study is limited to data collected during a single breeding season that coincided with a surprising superabundance of *C. guttivitta* (saddled prominent moth, Notodontidae; see below) relative to typical years at Hubbard Brook (Holmes & Schultz, 1988; Reynolds et al., 2007) and that warblers might select other Lepidoptera species in the absence of *C. guttivitta*. However, the patterns we describe from this single breeding season provide a framework for understanding the capacity of black‐throated blue warblers to respond to the potential effects of changing seasonality on early‐ and late‐season insect availability and identify periods when migratory birds are most vulnerable to change. For example, the composition and richness of migratory bird diets are hypothesized to change over time with shifts in the phenology of Lepidoptera prey resulting from warming spring and autumn temperatures (e.g., Samplonius et al., 2016). Moreover, migratory birds are hypothesized to exhibit specialist foraging strategies at high prey availability and become more generalist foragers at low prey availability (e.g., Tucker et al., 2019). By incorporating diet studies using the DNA metabarcoding approach and prey surveys into long‐term monitoring of migratory bird populations, such as ours, we can explore hypotheses examining adaptive population responses to climate‐induced changes in prey phenology and abundance.

### 4.2 Diet composition

Fecal DNA metabarcoding (*n* = 87) revealed a much higher richness and diversity of arthropod taxa in the diets of black‐throated blue warblers (Table S7) than previous observational studies of their stomach contents (*n* = 58 samples) and prey consumed during foraging observations (*n* = 109 prey taken) at Hubbard Brook (Robinson & Holmes, 1982). This difference in results from these approaches is consistent with diet studies of other bird species demonstrating an increase in the number of prey species and genera detected using molecular approaches compared to observational approaches (Alonso et al., 2014; Chung et al., 2021; Oehm et al., 2016; Thalinger et al., 2022). Past observational diet studies of black‐throated blue warblers were conducted from mid‐June to mid‐July (corresponding to our mid‐survey period) from 1974 to 1979 during years with no insect outbreaks. Our fecal DNA metabarcoding results were more like the diet composition described from foraging observations than from stomach contents. For example, Robinson and Holmes (1982) found that the frequency of occurrence of Lepidoptera (butterflies and moths) comprised 28% of stomach contents (all larvae) and 95% of observations of prey consumed (81% adults, 14% larvae). The frequency of occurrence of adult Diptera (flies) constituted 12% of stomach contents and 2% of observations of prey taken. We found a similarly high frequency of occurrence of Lepidoptera (100%) in fecal samples (arthropod life stage unknown) but a high occurrence of flies (94.3% Diptera). Coleoptera (beetles) were present in 50% of stomach contents but were not observed as prey captured during foraging observations and occurred in low frequency in fecal samples (28.7%). The greatest disparity between fecal DNA metabarcoding results and diets characterized by both stomach contents and foraging observations was the high occurrence of spiders, especially during the early‐ and mid‐survey periods (Araneae, 98.9%), which were not detected in stomach contents and constituted only 4% of foraging observations. The lower occurrence of prey such as spiders and Diptera in diets described from observational approaches reflects the lower detectability of softer‐bodied prey and smaller prey items in stomach contents and during foraging observations compared to DNA metabarcoding (Hoenig et al., 2022; Stillman et al., 2022).

### 4.3 Seasonal diet flexibility

Diet varied over the season, with the highest prey species richness and diversity occurring during the early season and the highest variance in diet composition during the late season. Dietary patterns reflected seasonal changes in insect availability and richness, which supported our hypothesis and indicated that black‐throated blue warblers can be flexible in their diets and take advantage of seasonal flushes of insect availability. For migratory species that rely on temporary pulses of prey availability, dietary flexibility is a key mechanism that may help birds respond to climate‐driven mismatches between breeding phenology and peak resource availability (Both et al., 2009, 2010; Burger et al., 2012). For instance, migratory wood warblers (*Phylloscopus sibilatrix*) breeding in European oak woodlands experience a pronounced seasonal peak in caterpillar biomass (Smith et al., 2011). By switching to other prey types during the breeding season, they avoided the negative fitness effects of phenological mismatch with their favored caterpillar prey (Mallord et al., 2017). Thus, insectivorous birds with a capacity for diet flexibility may experience fewer negative impacts from shifts in prey phenology (e.g., Dunn et al., 2011; Reed et al., 2013).

Black‐throated blue warblers do not depend on a single prey source during the breeding season and exhibit plasticity in their breeding phenology in response to changes in the emergence of leaves and their caterpillar prey (Lany et al., 2016). They have advanced both their arrival to Hubbard Brook and their timing of breeding in response to warmer springs and earlier leaf out (Lany et al., 2016). This has extended their breeding season with fitness advantages for early breeders that raise second broods (Townsend et al., 2013). Higher diet richness and diversity when black‐throated blue warbler pairs initiate their first clutches suggest diet flexibility early in the season might be necessary and especially important. Upon arrival at Hubbard Brook, adults must restore their energy reserves from migration and prepare to breed. A diverse diet could buffer black‐throated blue warblers from changes in early‐season insect availability. Likewise, the greater variance in diet composition when black‐throated blue warbler pairs are raising late broods suggests that this species might exhibit the flexibility in their diets to respond positively to changes in late‐season insect availability. However, the long‐term fitness consequences of longer green seasons for insectivorous birds are unclear without knowing how insect populations will respond. In particular, early‐ and late‐season caterpillar availability is expected to change with shifts in leaf phenology that affect prey emergence, migration, number of generations, and diapause (Gallinat et al., 2015). The forested habitat at our study site supports a substantial diversity of Lepidoptera taxa (Stange et al., 2011). However, Lepidoptera biomass lacks single predictable seasonal peaks and can vary by 20‐fold over the years (Lany et al., 2016; Reynolds et al., 2007). High seasonal unpredictability and inter‐annual variability in diverse caterpillar prey resources at Hubbard Brook might be selected for diet flexibility in black‐throated blue warblers.

### 4.4 Dietary overlap across elevation zones and among age groups

Diet composition and diversity did not differ significantly across elevation zones. Although our study plot encompasses a 2‐week difference in green season length across an elevation gradient, the difference from low to high elevations (380–740 m; 360 m) was potentially not great enough to alter the composition of the arthropod community and detect significant differences in arthropod composition or diversity in diets. Studies conducted at larger scales in elevation change than ours have shown significant variation in arthropod abundance and community composition across elevations associated with environmental differences (e.g., climate, seasonality, habitat, and predators) (Fernandez‐Conradi et al., 2020; Supriya et al., 2019; Uhey et al., 2022). Few studies have examined the diets of insectivorous birds across elevations to compare to our results. However, in a study of blue tits (*Cyanistes caeruleus*) conducted across an elevation gradient in Scotland, diet richness did not vary with elevation (Shutt et al., 2020). Birds that are diet generalists are predicted to respond to climate change by expanding their ranges because they can exploit novel prey (Buckley & Kingsolver, 2012). Numerous insectivorous montane birds have shifted their breeding distributions upslope in response to increasing temperatures and downslope in response to increasing precipitation from climate change (e.g., DeLuca & King, 2017; Freeman et al., 2018; Rodenhouse et al., 2008; Tatenhove et al., 2019). An understanding of a species' diet flexibility across broader elevation ranges relative to insect availability is needed to assess a population's vulnerability to potential declines linked to elevation shifts in their breeding distributions (Auer & King, 2014; Sam et al., 2017).

Dietary overlap among warbler age groups was high, but we found significant differences in diet composition between juveniles (HY) and adults (SY and ASY). Nonetheless, this result must be interpreted with caution based on a limited sample size of juvenile birds (*n* = 5). Dietary differences between these age groups contributed to diet variation in the late survey period (when all HY birds were caught). However, when juveniles were removed from the analysis, diet composition remained different in the late survey period (Table S8). Juvenile diets contained a higher proportion of Psocodea (lice), Neuroptera (lacewings), Tromidiformes (mites), and Opiliones (harvestman spiders) than adults (Table S9). Nestlings are more susceptible to lice and mites than adults because the confined environment of the nest provides a favorable habitat for these ectoparasites to thrive (Proctor & Owens, 2000). This might explain the higher detection of lice and mites in the few samples we collected from juveniles a few weeks after fledging, either from consumption or contamination from the nest environment. Adult diets contained a higher proportion of Coleoptera (beetles) and Plecoptera (stoneflies) than juveniles (Table S9). Subtle diet differences between juveniles and adults might occur if they have different foraging strategies, partition foraging space to reduce resource competition, or if adults specialize in preferred prey because of higher foraging proficiency (Davies et al., 2022). A larger sample of juveniles will be needed to explore these age‐related patterns further.

### 4.5 Importance of different prey species

Fecal DNA metabarcoding allowed us to examine the diets of black‐throated blue warblers at a specificity that is impossible to replicate in observational studies (Hoenig et al., 2022). We identified 242 species in 87 fecal samples, including a high percentage of rare prey items (192 species; 48.6% of taxa detected in only one sample). We analyzed diets using the presence–absence data to reduce the effect of sequencing bias (Deagle et al., 2019; Jusino et al., 2019), but we note that this approach omits information about the relative abundance of prey items in individual samples. However, when calculated across multiple samples, information on the frequency of occurrence of prey taxa provides functional inference on prey importance (Deagle et al., 2019). By matching sampled diets to field surveys of prey availability, these data can help us to understand how the availability or declines of specific prey at different times of the season might affect the fitness of birds.

Black‐throated blue warblers forage primarily for Lepidoptera (Robinson & Holmes, 1982) and mainly feed their nestlings caterpillars (60–87% of prey delivered) (Goodbred & Holmes, 1996). Thus, we expected their diets to be comprised of mostly Lepidoptera. Indeed, all sampled individuals included Lepidoptera prey in their diet, representing 21 separate families. *C. guttivitta* (saddled prominent moth, Notodontidae) occurred in the highest frequency (79%) of samples across survey periods (early: 83.3%, mid: 100%, and late: 87.5%). Outbreaks of this native species occur every 10 years, causing widespread defoliation in hardwood forests (Rush & Allen, 1987). This species feeds on the foliage of a variety of trees and shrubs but prefers American beech, sugar maple, and yellow birch (Johnson & Lyon, 1991), the dominant tree species at Hubbard Brook (Schwarz et al., 2003; van Doorn et al., 2011). As a result, populations build rapidly in forests with a high proportion of these tree species for 1–3 years, followed by a collapse (Rush & Allen, 1987). The extended period of adult emergence, oviposition, and larval development results in several stages of the saddled prominent moth being present simultaneously (Wagner, 2005). We anticipated high detections of this species in their diet, given their prevalence on caterpillar surveys in both 2020 and 2021. However, the saddled prominent moth is not typically present in high abundance at Hubbard Brook (Holmes & Schultz, 1988; Reynolds et al., 2007), and other Lepidoptera species might be selected in years without *C*. *guttivitta* outbreaks.

Black‐throated blue warblers selected and consumed Lepidoptera (life stage unknown) and spiders throughout the breeding season. However, the frequency of occurrence of spiders in diets was higher early in the season when caterpillar abundance was low. Caterpillars are nutrient‐rich and contain high carotenoid content (Arnold et al., 2010), which increases antioxidant capacity in birds and helps to protect them from oxidative damage caused by the metabolic demands of growth and reproduction (Metcalfe & Monaghan, 2013; Skrip & McWilliams, 2016). Spiders have high nutritional value as they contain high concentrations of dietary taurine (Ramsay & Houston, 2003), an amino acid that increases antioxidant defense and neurological function in birds (Han et al., 2020). Taurine is also important for the development and growth of birds, particularly during early life stages (Arnold et al., 2007; Ramsay & Houston, 2003). The timing of breeding can affect the nutritional quality of both caterpillars and spiders consumed by adults and fed to nestlings (Arnold et al., 2010). Seasonal selection for certain Lepidoptera prey might reflect differences in nutritional quality and value to breeding adults.

### 4.6 Prey selection and avoidance

Overall, seasonal variation in diet composition tended to track temporal changes in insect availability at Hubbard Brook. This variation in diet demonstrates that black‐throated blue warblers can respond to fluctuating prey availability and take advantage of a diverse and unpredictable food supply. Insect communities are subject to rapid changes in response to environmental change (Tallamy & Shriver, 2021). Black‐throated blue warblers opportunistically consumed certain prey items when more available, suggesting their diets will likely track changes in the insect community. Other DNA metabarcoding studies of generalist insectivores have shown similar dietary flexibility reflecting changes in the seasonal availability of insect prey, for example, barn swallows, *Hirundo rustica* (McClenaghan et al., 2019), blue tits (Shutt et al., 2020), and Eurasian reed warblers, *Acrocephalus scirpaceus* (Davies et al., 2022). These studies highlight the importance of dietary flexibility in population responses to climate change and the need for long‐term monitoring of changes in diet composition relative to the abundance and phenology of insects, especially early and late in the season.

Despite the general pattern of diets tracking availability, our results from two separate field sampling efforts highlight several instances where warblers exhibit apparent selection or avoidance of certain prey. First, we designed our caterpillar surveys to examine seasonal changes in the biomass of the primary Lepidoptera families available to foliage‐gleaning warblers at Hubbard Brook and consumed by black‐throated blue warblers during foraging observations (Holmes et al., 1979; Holmes & Schultz, 1988). We found evidence for prey selection and avoidance of specific Lepidopteran families in the early and mid‐survey periods but not in the late‐survey period. In the early and mid‐survey periods, black‐throated blue warblers showed selection for Notodontids when the availability of these prey (as caterpillars) was relatively low (Figures 3 and 5) and avoidance of Noctuidae when their availability (as caterpillars) was relatively high (Figures 3 and 5). Second, we used Malaise traps to measure the capture frequency of 9 insect taxa to examine seasonal changes in the availability of flying insects (Rodenhouse & Holmes, 1992). We found that diet composition tracked the availability of flying insect prey in general (Figure S3), but taxon‐specific patterns of selection or avoidance were also evident (Figure 5). Prey avoidance could represent taxa not consumed because of toxins that make prey unpalatable, size limitations, or low encounter rates if prey activity and foraging occur at different times (i.e., diurnal vs. nocturnal) (Cooper & Whitmore, 1990; Savage et al., 2010). The presence at Hubbard Brook of some unpalatable Noctuid caterpillars, such as the Acronictinae subfamily, is one possibility for the avoidance of Noctuidae prey by warblers. Arthropods (all life stages) vary in size, mobility, moisture content, and nutritional content (e.g., fats, proteins, and carotenoids) (Razeng & Watson, 2015; Reeves et al., 2021; Senti & Gifford, 2024), likely contributing to prey selection. Future studies that simultaneously collect data on diet and insect availability should consider incorporating analysis of prey nutritional content to examine how the macronutrient and micronutrient values of arthropod groups vary seasonally.

No sampling method can effectively capture all arthropod taxa identified in bird diets. Malaise traps are effective at sampling some Hemipteran families, including Miridae (plant bugs) and Cicadellidae (leafhoppers) (Montgomery et al., 2021), the most represented Hemipteran families in warbler diets (Table 2). However, Malaise traps were not designed to sample some taxa that showed a high frequency of occurrence in warbler diets, including beetles (Coleoptera) and spiders (Araneae) that exhibit seasonal behavioral shifts that can affect sampling efficiency (Montgomery et al., 2021; Oxbrough et al., 2010). In analyses comparing prey availability to warbler diets, we removed taxa with <15 individuals captured over the study period and arthropod taxa potentially affected by the sampling bias of Malaise traps. Differences between diet data and insect availability could arise from species‐level biases in Malaise capture rates. However, after aggregating insect data into broader taxonomic groups for analysis, we have no reason to speculate that this occurred systematically to generate false signatures of prey selection or avoidance.

### 4.7 Climate change implications

Depending on their life histories and foraging behavior, changing seasonality might affect species' diets. Multiple‐brooded species, like black‐throated blue warblers, might benefit from longer green seasons by increasing their diet breadth and rearing additional broods (Halupka et al., 2021; Townsend et al., 2013), whereas single‐brooded species might have less ecological flexibility to adaptively respond to changes in late season food availability (Halupka & Halupka, 2017). Birds that forage primarily from the green “foliar” food web might also broaden their diets in response to longer green seasons if changes in leaf phenology affect insect availability. In contrast, birds that forage from the more stable brown “forest floor” food web might have less variable diets due to differences in the extent of warming‐induced trophic mismatches in green and brown food webs (Thakur, 2020). To better understand how bird communities might respond to longer green seasons, we suggest that future studies examine species' diets that differ in their propensity to double brood and their reliance on the green and brown food webs. Determining how species' diet flexibility differs relative to variation in these food webs would help predict which species might be more resilient to climate‐induced changes in their food supply.

Our results also highlighted that certain arthropod prey were especially important during critical periods of the breeding season when birds were initiating their first broods and provisioning nestlings and fledglings from late broods. Food is a limiting factor for breeding productivity (Rodenhouse & Holmes, 1992), with late‐season food availability being the best predictor of double brooding in black‐throated blue warblers (Kaiser et al., 2015; Nagy & Holmes, 2005a, 2005b). Declines in Lepidoptera due to climate change (Conrad et al., 2006; Sánchez‐Bayo & Wyckhuys, 2019; Thomas et al., 2004) might increase the time and energy spent searching for prey or prevent black‐throated blue warblers from raising second broods if food supplies are insufficient.

## 5 CONCLUSIONS

Seasonal comparisons of the occurrence of arthropod prey in diets using fecal DNA metabarcoding relative to prey availability revealed seasonal changes in black‐throated blue warbler diets that tracked pulses in arthropod prey availability and prey selectivity early and late in the season when birds are more vulnerable to changes in insect availability. Insectivorous birds are being impacted by insect population declines worldwide (Tallamy & Shriver, 2021), and diet flexibility might help shield them from some of the adverse effects of these insect declines. DNA metabarcoding is a powerful tool to assess avian diets at levels of specificity that are not achievable by other diet analysis methods (Hoenig et al., 2022). Long‐term studies of migratory birds and insect communities incorporating diet analyses using DNA metabarcoding will be necessary to test hypotheses about adaptive responses to changing seasonality and phenology across trophic scales in the green and brown food webs.

## AUTHOR CONTRIBUTIONS

**Sara A. Kaiser:** Conceptualization (lead); data curation (equal); funding acquisition (equal); investigation (supporting); methodology (equal); project administration (lead); resources (equal); supervision (equal); validation (equal); writing – original draft (equal); writing – review and editing (lead). **Gemma V. Clucas:** Data curation (equal); formal analysis (equal); investigation (equal); methodology (equal); project administration (supporting); supervision (equal); validation (equal); writing – original draft (equal); writing – review and editing (equal). **T. Scott Sillett:** Funding acquisition (supporting); project administration (supporting); resources (equal); writing – review and editing (supporting). **Lindsey E. Forg:** Conceptualization (supporting); data curation (equal); formal analysis (supporting); funding acquisition (equal); investigation (equal); methodology (supporting); writing – original draft (equal); writing – review and editing (equal). **Andrew N. Stillman:** Data curation (equal); formal analysis (lead); methodology (equal); validation (equal); visualization (lead); writing – original draft (supporting); writing – review and editing (equal). **John F. Deitsch:** Data curation (supporting); investigation (equal); writing – review and editing (supporting).

## CONFLICT OF INTEREST STATEMENT

The authors declare we have no competing interests.

## Supporting information


Data S1:

## Data Availability

Statistical analyses reported in this article can be reproduced using the datasets and code provided in the Environmental Data Initiative repository: https://doi.org/10.6073/pasta/2091305bf6653a8aaa26964cf1470740. All metabarcoding data associated with this work are available at the NCBI Sequence Read Archive (Kaiser et al. 2024).
