# Supplementary material for: Black‐throated blue warblers (Setophaga caerulescens) exhibit diet flexibility and track seasonal changes in insect availability
Source: Ecol Evol. 2024 Sep 20;14(9):e70340. doi: 10.1002/ece3.70340 (PMC11413564; doi:10.1002/ece3.70340)
Supplement: Supplementary file 1 — Data S1: [file ECE3-14-e70340-s001.docx]

**Supplementary Material** from “Black-throated blue warblers (*Setophaga caerulescens*) exhibit diet flexibility and track seasonal changes in insect availability”

Sara A. Kaiser, Lindsey E. Forg, Andrew N. Stillman, John F. Deitsch, T. Scott Sillett, and Gemma V. Clucas

Corresponding author: Sara Kaiser, [sak275@cornell.edu](mailto:sak275@cornell.edu); 159 Sapsucker Woods Rd, Center for Biodiversity Sciences, Cornell Lab of Ornithology, Cornell University, Ithaca, NY, 14850, USA

**A. Supplementary Tables**

| **TABLE S1.** Distribution of Black-throated blue warbler fecal samples by elevation zone, survey period, age group, and sex collected at the Hubbard Brook Experimental Forest, New Hampshire, USA (total = 99 fecal samples). | | |
| --- | --- | --- |
| **Variable** | **Group** | ***n*** |
| Survey period | Early | 29 |
|  | Mid | 34 |
|  | Late | 36 |
|  |  |  |
| Elevation zone | Low | 33 |
|  | Mid | 32 |
|  | High | 34 |
|  |  |  |
| Age class | HY | 5 |
|  | SY | 57 |
|  | ASY | 37 |
|  |  |  |
| Sex | Male | 66 |
|  | Female | 33 |

| **TABLE S2.** Family-specific biomass (mg) of three focal Lepidopteran families surveyed on transects at three elevation zones during three survey periods at the Hubbard Brook Experimental Forest, New Hampshire, USA. | | | | | |
| --- | --- | --- | --- | --- | --- |
| **Elevation zone ^a^** | **Survey Period ^b^** | **Geometridae** | **Noctuidae** | **Notodontidae** | **Total** |
| Low | Early | 7.93 | 50.79 | 0.03 | 58.75 |
| Low | Mid | 155.99 | 71.40 | 71.08 | 298.47 |
| Low | Late | 920.20 | 0.07 | 905.60 | 1825.87 |
| Mid | Early | 21.17 | 1.36 | 0.00 | 22.53 |
| Mid | Mid | 233.64 | 24.68 | 99.99 | 358.31 |
| Mid | Late | 562.74 | 6.06 | 849.50 | 1418.3 |
| High | Early | 9.43 | 29.03 | 0.00 | 38.46 |
| High | Mid | 123.00 | 19.40 | 116.69 | 259.09 |
| High | Late | 1710.86 | 32.40 | 935.42 | 2678.68 |
| ^a^ Elevation zones = Low: 380–499 m, Mid: 500–599 m, High: 600–740 m | | | | | |
| ^b^ Survey periods = Early: 15 May–15 Jun, Mid: 16 Jun–15 Jul, Late: 16 Jul–15 Aug | | | | | |

| **TABLE S3.** Number of individuals >4 mm in length in each taxonomic group sampled in Malaise traps at three elevation zones during three survey periods at the Hubbard Brook Experimental Forest, New Hampshire, USA. | | | | | | | | | | | |
| --- | --- | --- | --- | --- | --- | --- | --- | --- | --- | --- | --- |
| **Elevation zone ^a^** | **Survey period ^b^** | **Plecoptera** | **Trichoptera** | **Diptera Tipulidae** | **Diptera**  **Rhagionidae** | **Hymenoptera**  **Non-Ichneumonids** | **Hymenoptera Ichneumonids** | **Panorpidae** | **Lepidoptera** | **Diptera Other** | **Total** |
| Low | Early | 1 | 23 | 13 | 5 | 3 | 21 | 5 | 31 | 71 | 173 |
| Low | Mid | 0 | 13 | 12 | 2 | 6 | 16 | 11 | 63 | 42 | 165 |
| Low | Late | 0 | 7 | 18 | 2 | 2 | 20 | 14 | 76 | 146 | 285 |
| Mid | Early | 1 | 6 | 35 | 9 | 5 | 47 | 1 | 48 | 108 | 260 |
| Mid | Mid | 1 | 5 | 19 | 1 | 3 | 53 | 3 | 61 | 41 | 187 |
| Mid | Late | 1 | 9 | 64 | 5 | 2 | 24 | 3 | 47 | 257 | 412 |
| High | Early | 8 | 24 | 74 | 12 | 4 | 35 | 3 | 58 | 97 | 315 |
| High | Mid | 2 | 9 | 10 | 0 | 4 | 53 | 6 | 87 | 70 | 241 |
| High | Late | 1 | 4 | 42 | 1 | 0 | 18 | 2 | 35 | 84 | 187 |
| ^a^ Elevation zones = Low: 380–499 m, Mid: 500–599 m, High: 600–740 m | | | | | | | | | | | |
| ^b^ Survey periods = Early: 15 May–15 Jun, Mid: 16 Jun–15 Jul, Late: 16 Jul–15 Aug | | | | | | | | | | | |

| **TABLE S4.** Frequency of occurrence (%) of all prey orders identified in the diets of black-throated blue warblers at the Hubbard Brook Experimental Forest, New Hampshire, USA. Total frequency is the percentage of the total number of samples across survey periods that included a given order. | | |
| --- | --- | --- |
| **Order** | **Description** | **Total** |
| Lepidoptera | butterflies and moths | 100 |
| Araneae | spiders | 98.9 |
| Diptera | true flies | 94.3 |
| Hemiptera | true bugs | 62.1 |
| Hymenoptera | sawflies, wasps, bees, and ants | 44.8 |
| Psocodea | barklice, booklice, parasitic lice | 34.5 |
| Coleoptera | beetles | 28.7 |
| Plecoptera | stoneflies | 17.2 |
| Neuroptera | lacewings, mantidflies, antlions, and their relatives | 4.6 |
| Sarcoptiformes | mites | 4.6 |
| Entomobryomorpha | springtails | 3.4 |
| Trombidiformes | mites | 3.4 |
| Opiliones | harvestman spiders | 2.3 |
| Poduromorpha | springtails | 2.3 |
| Blattodea | cockroaches and termites | 1.1 |
| Julida | millipedes | 1.1 |
| Mesostigmata | mites | 1.1 |

| **TABLE S5.** Pairwise comparisons of the mean difference in multivariate dispersion between survey periods and their 95% confidence intervals (CI). | | | | |
| --- | --- | --- | --- | --- |
| **Survey period comparison** | **Mean difference between survey periods** | **Lower CI** | **Upper CI** | ***p*-value** |
| Late-Early | 0.035 | 0.001 | 0.069 | 0.042 |
| Mid-Early | -0.016 | -0.049 | 0.018 | 0.519 |
| Mid-Late | -0.051 | -0.082 | -0.019 | <0.001 |

| **TABLE S6.** Frequency of occurrence (%) of all prey families identified in the diets of black-throated blue warblers in each survey period at the Hubbard Brook Experimental Forest, New Hampshire, USA. Taxonomy was assigned from the Barcode of Life Database (BOLD). Each row represents a different family. Total frequency is the percentage of the total number of samples across survey periods that included a given family. | | | | | | | |
| --- | --- | --- | --- | --- | --- | --- | --- |
|  |  |  |  |  | **Survey Period** | | |
|  |  |  |  | **Total** | **early** | **mid** | **late** |
| **Class** | **Order** | **Family** | **Common Name** | ***n* = 87** | ***n* = 24** | ***n* = 31** | ***n* = 32** |
| Insecta | Lepidoptera | Notodontidae | Prominent Moths | 79.0 | 83.3 | 100 | 87.5 |
| Insecta | Lepidoptera | Geometridae | Geometer Moths | 71.0 | 83.3 | 74.2 | 87.5 |
| Arachnida | Araneae | Theridiidae | Cobweb Spiders | 68.0 | 91.7 | 93.5 | 53.1 |
| Arachnida | Araneae | Araneidae | Orbweavers | 53.0 | 79.2 | 64.5 | 43.8 |
| Arachnida | Araneae | Philodromidae | Running Crab Spiders | 53.0 | 83.3 | 71.0 | 34.4 |
| Insecta | Lepidoptera | Tortricidae | Tortricid Leafroller Moths | 42.0 | 62.5 | 67.7 | 18.8 |
| Arachnida | Araneae | Linyphiidae | Sheetweb Dwarf Weavers | 41.0 | 54.2 | 32.3 | 56.2 |
| Insecta | Hemiptera | Miridae | Plant Bugs | 36.0 | 25 | 74.2 | 21.9 |
| Insecta | Diptera | Rhagionidae | Snipe Flies | 33.0 | 62.5 | 22.6 | 34.4 |
| Arachnida | Araneae | Dictynidae | Meshweavers | 32.0 | 50.0 | 41.9 | 21.9 |
| Insecta | Hymenoptera | Ichneumonidae | Ichneumonid Wasps | 30.0 | 29.2 | 29.0 | 43.8 |
| Insecta | Diptera | Cecidomyiidae | Gall and Forest Midges | 29.0 | 37.5 | 32.3 | 31.2 |
| Insecta | Diptera | Tachinidae | Bristle Flies | 24.0 | 16.7 | 29.0 | 34.4 |
| Insecta | Diptera | Mycetophilidae | Fungus Gnats | 22.0 | 8.3 | 16.1 | 46.9 |
| Arachnida | Araneae | Tetragnathidae | Long-jawed Orbweavers | 22.0 | 50.0 | 22.6 | 9.4 |
| Insecta | Hemiptera | Cicadellidae | Typical Leafhoppers | 20.0 | 20.8 | 22.6 | 25.0 |
| Insecta | Lepidoptera | Gelechiidae | Twirler Moths | 20.0 | 33.3 | 25.8 | 12.5 |
| Insecta | Diptera | Chironomidae | Non-biting Midges | 18.0 | 33.3 | 16.1 | 15.6 |
| Insecta | Diptera | Limoniidae | Limoniid Crane flies | 17.0 | 25.0 | 22.6 | 12.5 |
| Insecta | Diptera | Sciaridae | Dark-winged Fungus Gnats | 17.0 | 25.0 | 9.7 | 25.0 |
| Insecta | Diptera | Keroplatidae | Predatory Fungus Gnats | 16.0 | 33.3 | 19.4 | 6.2 |
| Insecta | Diptera | Culicidae | Mosquitoes | 15.0 | 41.7 | 12.9 | 3.1 |
| Insecta | Lepidoptera | Erebidae | Underwing, Tiger, Tussock, and Allied Moths | 14.0 | 25.0 | 12.9 | 12.5 |
| Insecta | Psocodea | Amphipsocidae | Hairy-winged Barklice | 13.0 | 0.0 | 32.3 | 9.4 |
| Insecta | Hymenoptera | Braconidae | Braconid Wasps | 13.0 | 20.8 | 22.6 | 3.1 |
| Insecta | Lepidoptera | Blastobasidae | Blastobasid Moths | 12.0 | 8.3 | 19.4 | 12.5 |
| Insecta | Lepidoptera | Depressariidae | Flat-bodied Moths | 12.0 | 4.2 | 35.5 | 0.0 |
| Insecta | Psocodea | Psocidae | Common Barklice | 12.0 | 4.2 | 6.5 | 28.1 |
| Insecta | Lepidoptera | Sphingidae | Sphinx Moths | 12.0 | 4.2 | 22.6 | 12.5 |
| Arachnida | Araneae | Clubionidae | Sac Spiders | 11.0 | 4.2 | 19.4 | 12.5 |
| Insecta | Plecoptera | Nemouridae | Forestflies | 11.0 | 20.8 | 16.1 | 3.1 |
| Insecta | Diptera | Empididae | Dance Flies | 10.0 | 25 | 12.9 | 0.0 |
| Insecta | Coleoptera | Elateridae | Click Beetles | 8.0 | 16.7 | 3.2 | 9.4 |
| Arachnida | Araneae | Theridiosomatidae | Ray Orbweavers | 8.0 | 8.3 | 3.2 | 15.6 |
| Insecta | Coleoptera | Cantharidae | Soldier Beetles | 7.0 | 0.0 | 3.2 | 18.8 |
| Insecta | Lepidoptera | Crambidae | Crambid Snout Moths | 7.0 | 8.3 | 9.7 | 6.2 |
| Insecta | Coleoptera | Curculionidae | True Weevils | 7.0 | 8.3 | 3.2 | 12.5 |
| Insecta | Diptera | Tipulidae | Large Crane Flies | 7.0 | 0.0 | 12.9 | 9.4 |
| Insecta | Diptera | Hybotidae | Hybotid Dance Flies | 6.0 | 0.0 | 12.9 | 6.2 |
| Insecta | Diptera | Pipunculidae | Big-headed Flies | 6.0 | 8.3 | 6.5 | 6.2 |
| Arachnida | Araneae | Agelenidae | Funnel Weavers | 5.0 | 0.0 | 0.0 | 15.6 |
| Insecta | Lepidoptera | Gracillariidae | Leaf Blotch Miner Moths | 5.0 | 16.7 | 3.2 | 0.0 |
| Insecta | Diptera | Muscidae | House Flies and Allies | 5.0 | 4.2 | 3.2 | 9.4 |
| Insecta | Hemiptera | Aphrophoridae | True Spittlebugs | 4.0 | 8.3 | 6.5 | 0.0 |
| Insecta | Coleoptera | Melandryidae | False Darkling Beetles | 4.0 | 12.5 | 3.2 | 0.0 |
| Insecta | Lepidoptera | Noctuidae | Cutworm Moths and Allies | 4.0 | 16.7 | 0.0 | 0.0 |
| Insecta | Hemiptera | Pentatomidae | Stink Bugs | 4.0 | 4.2 | 6.5 | 3.1 |
| Insecta | Psocodea | Peripsocidae | Stout Barklice | 4.0 | 0.0 | 6.5 | 6.2 |
| Insecta | Lepidoptera | Psychidae | Bagworm Moths | 4.0 | 0.0 | 12.9 | 0.0 |
| Insecta | Coleoptera | Staphylinidae | Rove Beetles | 4.0 | 8.3 | 3.2 | 3.1 |
| Insecta | Hemiptera | Aphididae | Aphids | 3.0 | 4.2 | 0.0 | 6.2 |
| Insecta | Diptera | Ceratopogonidae | Biting Midges | 3.0 | 0.0 | 0.0 | 9.4 |
| Insecta | Neuroptera | Hemerobiidae | Brown Lacewings | 3.0 | 4.2 | 6.5 | 0.0 |
| Insecta | Lepidoptera | Lasiocampidae | Lappet Moths | 3.0 | 4.2 | 6.5 | 0.0 |
| Insecta | Plecoptera | Leuctridae | Rolled-winged Stoneflies | 3.0 | 12.5 | 0.0 | 0.0 |
| Insecta | Hemiptera | Reduviidae | Assassin Bugs | 3.0 | 8.3 | 0.0 | 3.1 |
| Insecta | Diptera | Simuliidae | Black Flies | 3.0 | 4.2 | 3.2 | 3.1 |
| Insecta | Diptera | Tabanidae | Horse and Deer Flies | 3.0 | 8.3 | 0.0 | 3.1 |
| Insecta | Diptera | Xylophagidae | Awl-Flies | 3.0 | 4.2 | 0.0 | 6.2 |
| Arachnida | Araneae | Amaurobiidae | Hackledmesh Weavers | 2.0 | 4.2 | 0.0 | 3.1 |
| Insecta | Diptera | Anthomyiidae | Root-maggot Flies | 2.0 | 0.0 | 6.5 | 0.0 |
| Insecta | Lepidoptera | Argyresthiidae | Shiny Headstand Moths | 2.0 | 0.0 | 3.2 | 3.1 |
| Insecta | Diptera | Bibionidae | March Flies | 2.0 | 8.3 | 0.0 | 0.0 |
| Arachnida | Opiliones | Caddidae | Harvestmen | 2.0 | 0.0 | 0.0 | 6.2 |
| Insecta | Psocodea | Caeciliusidae | Lizard Barklice | 2.0 | 0.0 | 3.2 | 3.1 |
| Insecta | Diptera | Clusiidae | Druid Flies | 2.0 | 4.2 | 3.2 | 0.0 |
| Insecta | Coleoptera | Coccinellidae | Lady Beetles | 2.0 | 4.2 | 0.0 | 3.1 |
| Insecta | Lepidoptera | Coleophoridae | Casebearing Moths | 2.0 | 4.2 | 3.2 | 0 |
| Arachnida | Araneae | Cybaeidae | Soft Spiders | 2.0 | 0.0 | 0.0 | 6.2 |
| Insecta | Diptera | Dolichopodidae | Long-legged Flies | 2.0 | 0.0 | 6.5 | 0.0 |
| Insecta | Diptera | Drosophilidae | Vinegar and Fruit Flies | 2.0 | 8.3 | 0.0 | 0.0 |
| Arachnida | Trombidiformes | Eupodidae | Trombidiform Mites | 2.0 | 4.2 | 0.0 | 3.1 |
| Collembola | Poduromorpha | Hypogastruridae | Hypogastrurid Springtails | 2.0 | 0.0 | 0.0 | 6.2 |
| Insecta | Lepidoptera | Incurvariidae | Leafcutter Moths | 2.0 | 8.3 | 0.0 | 0.0 |
| Insecta | Diptera | Iteaphila group | Dance Flies | 2.0 | 8.3 | 0.0 | 0.0 |
| Insecta | Diptera | Lauxaniidae | Lauxaniid Flies | 2.0 | 0.0 | 6.5 | 0.0 |
| Insecta | Psocodea | Lepidopsocidae | Scaly-winged Barklice | 2.0 | 0.0 | 6.5 | 0.0 |
| Insecta | Psocodea | Myopsocidae | Mouse-like Barklice | 2.0 | 0.0 | 0.0 | 6.2 |
| Arachnida | Sarcoptiformes | Peloppiidae | Beetle Mites | 2.0 | 0.0 | 0.0 | 6.2 |
| Insecta | Hymenoptera | Pergidae | Pergid Sawflies | 2.0 | 4.2 | 0.0 | 3.1 |
| Insecta | Coleoptera | Tenebrionidae | Darkling Beetles | 2.0 | 8.3 | 0.0 | 0.0 |
| Arachnida | Araneae | Thomisidae | Crab Spiders | 2.0 | 4.2 | 3.2 | 0.0 |
| Insecta | Lepidoptera | Tineidae | Fungus Moths, Clothes Moths, and Allies | 2.0 | 0.0 | 3.2 | 3.1 |
| Insecta | Diptera | Agromyzidae | Leaf-miner Flies | 1.0 | 4.2 | 0.0 | 0.0 |
| Arachnida | Mesostigmata | Ameroseiidae |  | 1.0 | 0.0 | 3.2 | 0.0 |
| Insecta | Hymenoptera | Argidae | Argid Sawflies | 1.0 | 0.0 | 0.0 | 3.1 |
| Insecta | Diptera | Asilidae | Robber Flies | 1.0 | 0.0 | 3.2 | 0.0 |
| Insecta | Lepidoptera | Autostichidae |  | 1.0 | 0.0 | 3.2 | 0.0 |
| Insecta | Diptera | Axymyiidae |  | 1.0 | 4.2 | 0.0 | 0.0 |
| Arachnida | Trombidiformes | Bdellidae | Snout Mites | 1.0 | 0.0 | 0.0 | 3.1 |
| Insecta | Coleoptera | Buprestidae | Jewel Beetles | 1.0 | 0.0 | 3.2 | 0.0 |
| Insecta | Diptera | Calliphoridae | Blow Flies | 1.0 | 0.0 | 0.0 | 3.1 |
| Insecta | Coleoptera | Carabidae | Ground Beetles | 1.0 | 0.0 | 0.0 | 3.1 |
| Insecta | Coleoptera | Cerambycidae | Longhorn Beetles | 1.0 | 0.0 | 3.2 | 0.0 |
| Insecta | Plecoptera | Chloroperlidae | Green Stoneflies | 1.0 | 4.2 | 0.0 | 0.0 |
| Insecta | Neuroptera | Coniopterygidae | Dusty-winged Lacewings | 1.0 | 0.0 | 0.0 | 3.1 |
| Arachnida | Sarcoptiformes | Cymbaeremaeidae | Beetle Mite Family | 1.0 | 0.0 | 0.0 | 3.1 |
| Insecta | Lepidoptera | Drepanidae | Hooktip and False Owlet Moths | 1.0 | 0.0 | 3.2 | 0.0 |
| Insecta | Hymenoptera | Dryinidae | Pincer Wasps | 1.0 | 0.0 | 0.0 | 3.1 |
| Insecta | Blattodea | Ectobiidae | Wood Cockroaches | 1.0 | 4.2 | 0.0 | 0.0 |
| Insecta | Hymenoptera | Formicidae | Ants | 1.0 | 4.2 | 0.0 | 0.0 |
| Insecta | Psocodea | Mesopsocidae | Middle Barklice | 1.0 | 0.0 | 0.0 | 3.1 |
| Insecta | Coleoptera | Mordellidae | Tumbling Flower Beetles | 1.0 | 0.0 | 0.0 | 3.1 |
| Insecta | Lepidoptera | Nepticulidae | Pygmy Eye-capped Moths | 1.0 | 4.2 | 0.0 | 0.0 |
| Insecta | Lepidoptera | Nolidae | Tufted Moths | 1.0 | 0.0 | 3.2 | 0.0 |
| Diplopoda | Julida | Parajulidae | Parajulid Millipedes | 1.0 | 4.2 | 0.0 | 0.0 |
| Arachnida | Sarcoptiformes | Parakalummidae | Beetle Mite Family | 1.0 | 4.2 | 0.0 | 0.0 |
| Insecta | Diptera | Pediciidae | Hairy-eyed Crane Flies | 1.0 | 0.0 | 3.2 | 0.0 |
| Insecta | Diptera | Phoridae | Humpbacked Flies | 1.0 | 0.0 | 3.2 | 0.0 |
| Arachnida | Sarcoptiformes | Phthiracaridae | Beetle Mite Family | 1.0 | 0.0 | 0.0 | 3.1 |
| Insecta | Hymenoptera | Platygastridae |  | 1.0 | 0.0 | 3.2 | 0.0 |
| Insecta | Coleoptera | Ptinidae | Deathwatch, Spider, and Wood-borer Beetles | 1.0 | 0.0 | 3.2 | 0.0 |
| Insecta | Coleoptera | Pyrochroidae | Fire-colored Beetles | 1.0 | 4.2 | 0.0 | 0.0 |
| Insecta | Hemiptera | Rhyparochromidae | Dirt-colored Seed Bugs | 1.0 | 4.2 | 0.0 | 0.0 |
| Arachnida | Araneae | Salticidae | Jumping Spiders | 1.0 | 4.2 | 0.0 | 0.0 |
| Insecta | Coleoptera | Scarabaeidae | Scarabs | 1.0 | 0.0 | 0.0 | 3.1 |
| Insecta | Diptera | Scathophagidae | Dung Flies | 1.0 | 0.0 | 0.0 | 3.1 |
| Insecta | Diptera | Syrphidae | Hover Flies | 1.0 | 0.0 | 3.2 | 0.0 |
| Arachnida | Trombidiformes | Tarsonemidae | Thread-footed Mites | 1.0 | 0.0 | 0.0 | 3.1 |

| **TABLE S7.** Frequency of occurrence (%) of all prey species identified in the diets of black-throated blue warblers in each survey period at the Hubbard Brook Experimental Forest, New Hampshire, USA. Taxonomy was assigned from the Barcode of Life Database (BOLD). Each row represents a different species, identified to the highest possible taxonomic rank. Total frequency is the percentage of the total number of samples across survey periods that included a given species. | | | | | | | | | |
| --- | --- | --- | --- | --- | --- | --- | --- | --- | --- |
|  |  |  |  |  |  |  | **Survey Period** | | |
|  |  |  |  |  |  | **Total** | **early** | **mid** | **late** |
| **Phylum** | **Class** | **Order** | **Family** | **Species** | **Common Name** | ***n* = 87** | ***n* = 24** | ***n* = 31** | ***n* = 32** |
| Arthropoda | Insecta | Lepidoptera | Notodontidae | *Cecrita guttivitta* | Saddled Prominent Moth | 79.0 | 83.3 | 100.0 | 87.5 |
| Arthropoda | Arachnida | Araneae | Theridiidae | *Theridion frondeum* | Eastern Long-legged Cobweaver | 53.0 | 70.8 | 93.5 | 21.9 |
| Arthropoda | Arachnida | Araneae | Philodromidae | *Philodromus rufus* | White-striped Running Crab Spider | 47.0 | 75.0 | 64.5 | 28.1 |
| Arthropoda | Insecta | Diptera |  |  | Insect spp. | 43.0 | 62.5 | 41.9 | 46.9 |
| Arthropoda | Insecta | Lepidoptera |  |  | Lepidoptera spp. | 33.0 | 54.2 | 51.6 | 12.5 |
| Arthropoda | Arachnida | Araneae | Araneidae | *Cyclosa conica* | Conical Trashline Orbweaver | 33.0 | 70.8 | 32.3 | 18.8 |
| Arthropoda | Arachnida | Araneae | Dictynidae | *Emblyna maxima* | Mesh Web Weaver Spider spp. | 30.0 | 45.8 | 41.9 | 18.8 |
| Arthropoda | Insecta | Lepidoptera | Geometridae | *Orthofidonia exornata* | Geometer Moth spp. | 28.0 | 4.2 | 25.8 | 59.4 |
| Arthropoda |  |  |  |  | Arthropod spp. | 28.0 | 37.5 | 6.5 | 53.1 |
| Arthropoda | Insecta | Lepidoptera | Tortricidae | *Pandemis lamprosana* | Woodgrain Leafroller Moth | 27.0 | 33.3 | 51.6 | 9.4 |
| Arthropoda | Insecta | Lepidoptera | Geometridae |  | Geometer Moth spp. | 26.0 | 4.2 | 25.8 | 53.1 |
| Arthropoda | Insecta | Diptera | Cecidomyiidae |  | Gall Midge | 25.0 | 37.5 | 29.0 | 21.9 |
| Arthropoda | Arachnida | Araneae | Tetragnathidae | *Tetragnatha shoshone* | Long-jawed Orbweaver spp. | 22.0 | 50.0 | 22.6 | 9.4 |
| Arthropoda | Insecta | Hemiptera | Miridae | *Deraeocoris grandis* | Plant bug | 20.0 | 16.7 | 48.4 | 3.1 |
| Arthropoda | Arachnida |  |  |  | Arachnid spp. | 20.0 | 12.5 | 12.9 | 40.6 |
| Arthropoda | Arachnida | Araneae | Linyphiidae | *Helophora insignis* | Sheetweb Spider sp. | 20.0 | 4.2 | 12.9 | 46.9 |
| Arthropoda | Arachnida | Araneae | Philodromidae | *Philodromus praelustris* | Running Crab Spider spp. | 20.0 | 37.5 | 19.4 | 15.6 |
| Arthropoda | Arachnida | Araneae | Araneidae | *Araneus saevus* | Fierce Orbweaver | 20.0 | 33.3 | 9.7 | 28.1 |
| Arthropoda | Arachnida | Araneae | Araneidae | *Araneus guttulatus* | Red-backed Orbweaver | 20.0 | 12.5 | 35.5 | 18.8 |
| Arthropoda | Arachnida |  |  |  | Arachnid spp. | 19.0 | 12.5 | 19.4 | 31.2 |
| Arthropoda | Arachnida | Araneae | Linyphiidae | *Pityohyphantes costatus* | Hammock Spider | 18.0 | 37.5 | 16.1 | 12.5 |
| Arthropoda | Insecta | Diptera | Rhagionidae | *Rhagio mystaceus* | Common Snipe Fly | 18.0 | 62.5 | 6.5 | 3.1 |
| Arthropoda | Arachnida | Araneae | Theridiidae | *Theridion murarium* | Fence Long-legged Cobweaver | 18.0 | 29.2 | 25.8 | 9.4 |
| Arthropoda | Insecta | Diptera | Keroplatidae |  | Predatory Fungus Gnat spp. | 16.0 | 33.3 | 19.4 | 6.2 |
| Arthropoda | Arachnida | Araneae | Theridiidae | *Parasteatoda tepidariorum* | Common House Spider | 16.0 | 12.5 | 12.9 | 28.1 |
| Arthropoda | Insecta | Lepidoptera | Geometridae | *Eutrapela clemataria* | Curved-toothed Geometer Moth | 15.0 | 8.3 | 29.0 | 12.5 |
| Arthropoda | Insecta | Psocodea | Amphipsocidae | *Polypsocus corruptus* | Corrupt Barklouse | 13.0 | 0.0 | 32.3 | 9.4 |
| Arthropoda | Insecta | Diptera | Tachinidae | *Blepharomyia tibialis* | Tachinid Fly spp. | 13.0 | 8.3 | 16.1 | 18.8 |
| Arthropoda | Insecta | Lepidoptera | Geometridae | *Eupithecia columbiata* | Pug moth spp. | 13.0 | 8.3 | 32.3 | 3.1 |
| Arthropoda | Insecta | Diptera | Rhagionidae | *Rhagio gracilis* | Snipe fly spp. | 12.0 | 0.0 | 3.2 | 34.4 |
| Arthropoda | Insecta | Lepidoptera | Depressariidae | *Psilocorsis reflexella* | Dotted Leaftier Moth | 12.0 | 4.2 | 35.5 | 0.0 |
| Arthropoda | Insecta | Psocodea | Psocidae | *Metylophorus novaescotiae* | Common Barklouse spp. | 12.0 | 4.2 | 6.5 | 28.1 |
| Arthropoda | Insecta | Diptera | Chironomidae |  | Non-biting Midge spp. | 12.0 | 20.8 | 12.9 | 9.4 |
| Arthropoda | Insecta | Lepidoptera | Geometridae | *Speranza pustularia* | Lesser Maple Spanworm Moth | 11.0 | 12.5 | 25.8 | 0.0 |
| Arthropoda | Insecta | Lepidoptera | Geometridae |  | Geometer Moth spp. | 11.0 | 4.2 | 6.5 | 25.0 |
| Arthropoda | Insecta | Lepidoptera | Geometridae | *Probole alienaria* | Friendly Probole Moth | 10.0 | 4.2 | 12.9 | 15.6 |
| Arthropoda | Insecta | Lepidoptera | Geometridae | *Lambdina fiscellaria* | Hemlock Looper Moth | 10.0 | 29.2 | 3.2 | 6.2 |
| Arthropoda | Insecta | Diptera | Culicidae | *Aedes communis* | Woodland Snow Pool Mosquito | 10.0 | 33.3 | 6.5 | 0.0 |
| Arthropoda | Insecta | Hemiptera | Miridae |  | Plant Bug spp. | 9.0 | 0.0 | 16.1 | 12.5 |
| Arthropoda | Arachnida | Araneae | Dictynidae | *Emblyna sublata* | Mesh web weaver spider spp. | 9.0 | 25.0 | 9.7 | 0.0 |
| Arthropoda | Insecta | Hemiptera | Cicadellidae |  | Typical Leafhopper spp. | 9.0 | 0.0 | 16.1 | 12.5 |
| Arthropoda | Insecta | Diptera | Mycetophilidae |  | Fungus Gnat spp. | 8.0 | 4.2 | 3.2 | 18.8 |
| Arthropoda | Arachnida | Araneae | Theridiosomatidae | *Theridiosoma gemmosum* | Common Eastern Ray Spider | 8.0 | 8.3 | 3.2 | 15.6 |
| Arthropoda | Insecta |  |  |  | Insect spp. | 8.0 | 4.2 | 3.2 | 18.8 |
| Arthropoda | Insecta | Lepidoptera | Tortricidae | *Olethreutes glaciana* | Leafroller Moth spp. | 8.0 | 4.2 | 19.4 | 3.1 |
| Arthropoda | Insecta | Plecoptera | Nemouridae | *Ostrocerca albidipennis* | Whitetailed Forestfly | 8.0 | 16.7 | 12.9 | 0.0 |
| Arthropoda | Arachnida | Araneae | Araneidae |  | Orbweaver spp. | 7.0 | 16.7 | 6.5 | 3.1 |
| Arthropoda | Insecta | Diptera | Limoniidae | *Elephantomyia westwoodi* | Limoniid Crane Fly | 7.0 | 4.2 | 12.9 | 6.2 |
| Arthropoda | Insecta | Diptera | Mycetophilidae |  | Fungus Gnat spp. | 7.0 | 4.2 | 9.7 | 9.4 |
| Arthropoda | Insecta | Lepidoptera | Blastobasidae | *Hypatopa simplicella* | Moth spp. | 7.0 | 0.0 | 19.4 | 3.1 |
| Arthropoda | Arachnida | Araneae | Clubionidae | *Clubiona canadensis* | Leaf-curling Sac spider spp. | 7.0 | 0.0 | 12.9 | 9.4 |
| Arthropoda | Insecta | Lepidoptera | Gelechiidae | *Carpatolechia belangerella* | Twirler Moth spp. | 7.0 | 8.3 | 12.9 | 3.1 |
| Arthropoda | Insecta | Diptera | Cecidomyiidae |  | Gall Midge spp. | 6.0 | 0.0 | 9.7 | 9.4 |
| Arthropoda | Insecta | Hymenoptera | Ichneumonidae |  | Ichneumonid Wasp spp. | 6.0 | 8.3 | 3.2 | 9.4 |
| Arthropoda | Insecta | Lepidoptera | Geometridae | *Melanolophia signataria* | Signate Melanolophia Moth | 6.0 | 0.0 | 16.1 | 3.1 |
| Arthropoda | Insecta | Coleoptera | Cantharidae | *Rhagonycha imbecillis* | Feeble Soldier Beetle spp. | 6.0 | 0.0 | 3.2 | 15.6 |
| Arthropoda | Insecta | Lepidoptera | Notodontidae | *Heterocampa biundata* | Wavy-lined Prominent | 6.0 | 4.2 | 12.9 | 3.1 |
| Arthropoda | Insecta | Lepidoptera | Geometridae | *Plagodis serinaria* | Lemon Plagodis Moth | 6.0 | 8.3 | 3.2 | 9.4 |
| Arthropoda | Insecta | Diptera | Culicidae |  | Mosquito spp. | 6.0 | 12.5 | 9.7 | 0.0 |
| Arthropoda | Insecta | Lepidoptera | Sphingidae | *Darapsa choerilus* | Azalea Sphinx | 6.0 | 4.2 | 12.9 | 3.1 |
| Arthropoda | Insecta | Hemiptera | Cicadellidae |  | Leafhopper spp. | 5.0 | 12.5 | 3.2 | 3.1 |
| Arthropoda | Insecta | Lepidoptera | Geometridae | *Ennomos subsignaria* | Elm Spanworm Moth | 5.0 | 12.5 | 6.5 | 0.0 |
| Arthropoda | Arachnida | Araneae | Agelenidae | *Agelenopsis utahana* | Grass Spider spp. | 5.0 | 0.0 | 0.0 | 15.6 |
| Arthropoda | Insecta | Diptera | Sciaridae | *Schwenckfeldina quadrispinosa* | Dark-winged Fungus Gnat spp. | 5.0 | 12.5 | 3.2 | 3.1 |
| Arthropoda | Insecta | Hymenoptera | Ichneumonidae | *Plectochorus iwatensis* | Ichneumonid Wasp spp. | 5.0 | 12.5 | 3.2 | 3.1 |
| Arthropoda | Insecta | Diptera | Empididae |  | Dance Fly spp. | 5.0 | 8.3 | 9.7 | 0.0 |
| Arthropoda | Insecta | Lepidoptera | Geometridae | *Ectropis crepuscularia* | Small Engrailed | 5.0 | 0.0 | 0.0 | 15.6 |
| Arthropoda | Insecta | Hymenoptera | Braconidae | *Peristenus braunae* | Braconid Wasp | 5.0 | 4.2 | 12.9 | 0.0 |
| Arthropoda | Insecta | Diptera | Limoniidae |  | Limoniid Crane Fly spp. | 5.0 | 16.7 | 3.2 | 0.0 |
| Arthropoda | Insecta | Diptera | Tipulidae | *Tipula hermannia* | Large Crane Fly spp. | 5.0 | 0.0 | 6.5 | 9.4 |
| Arthropoda | Insecta | Coleoptera | Elateridae | *Dalopius fuscipes* | Click Beetle spp. | 5.0 | 12.5 | 3.2 | 3.1 |
| Arthropoda | Insecta | Hymenoptera | Ichneumonidae |  | Ichneumonid Wasp spp. | 5.0 | 0.0 | 6.5 | 9.4 |
| Arthropoda | Arachnida | Araneae |  |  | Spider spp. | 5.0 | 8.3 | 6.5 | 3.1 |
| Arthropoda | Insecta | Lepidoptera | Geometridae | *Plagodis alcoolaria* | Hollow-spotted Plagodis Moth | 5.0 | 8.3 | 0.0 | 9.4 |
| Arthropoda | Insecta | Coleoptera | Curculionidae | *Strophosoma fulvicorne* | Broad-nosed Weevil spp. | 5.0 | 4.2 | 3.2 | 9.4 |
| Arthropoda | Insecta | Diptera | Mycetophilidae |  | Fungus Gnat spp. | 5.0 | 0.0 | 0.0 | 15.6 |
| Arthropoda | Insecta | Lepidoptera | Erebidae | *Idia rotundalis* | Rotund Idia Moth | 4.0 | 4.2 | 0.0 | 9.4 |
| Arthropoda | Insecta | Plecoptera |  |  | Stonefly spp. | 4.0 | 16.7 | 0.0 | 0.0 |
| Arthropoda | Insecta | Lepidoptera | Psychidae | *Psyche casta* | Common Bagworm Moth | 4.0 | 0.0 | 12.9 | 0.0 |
| Arthropoda | Insecta | Lepidoptera | Tortricidae | *Acleris cervinana* | Leafroller Moth spp. | 4.0 | 16.7 | 0.0 | 0.0 |
| Arthropoda | Insecta | Coleoptera | Melandryidae | *Symphora flavicollis* | False Darkling Beetle spp. | 4.0 | 12.5 | 3.2 | 0.0 |
| Arthropoda | Insecta | Diptera | Rhagionidae | *Chrysopilus quadratus* | Quadrate Snipe Fly | 4.0 | 4.2 | 9.7 | 0.0 |
| Arthropoda | Insecta | Hymenoptera | Ichneumonidae | *Dusona vitticollis* | Ichneumonid Wasp spp. | 4.0 | 0.0 | 0.0 | 12.5 |
| Arthropoda | Insecta | Diptera | Mycetophilidae | *Exechia frigida* | Fungus Gnat spp. | 4.0 | 0.0 | 0.0 | 12.5 |
| Arthropoda | Insecta | Diptera | Sciaridae |  | Dark-winged Fungus Gnat spp. | 4.0 | 4.2 | 0.0 | 9.4 |
| Arthropoda | Insecta | Lepidoptera | Sphingidae | *Amorpha juglandis* | Walnut Sphinx | 4.0 | 0.0 | 9.7 | 3.1 |
| Arthropoda | Insecta | Psocodea | Peripsocidae | *Peripsocus subfasciatus* | Stout Barklice spp. | 4.0 | 0.0 | 6.5 | 6.2 |
| Arthropoda | Insecta | Hemiptera | Pentatomidae | *Podisus brevispinus* | Stink Bug spp. | 4.0 | 4.2 | 6.5 | 3.1 |
| Arthropoda | Insecta | Hemiptera | Cicadellidae |  | Typical Leafhopper spp. | 4.0 | 8.3 | 6.5 | 0.0 |
| Arthropoda | Insecta | Hymenoptera | Ichneumonidae |  | Ichneumonid Wasp spp. | 4.0 | 0.0 | 3.2 | 9.4 |
| Arthropoda | Insecta | Lepidoptera | Noctuidae | *Orthosia rubescens* | Ruby Quaker | 4.0 | 16.7 | 0.0 | 0.0 |
| Arthropoda | Insecta | Diptera | Tachinidae |  | Bristle Fly spp. | 4.0 | 0.0 | 0.0 | 12.5 |
| Arthropoda | Insecta | Hemiptera | Aphrophoridae | *Aphrophora cribrata* | Pine Spittlebug | 4.0 | 8.3 | 6.5 | 0.0 |
| Arthropoda | Insecta | Diptera | Limoniidae | *Limonia indigena* | Limoniid Cranefly spp. | 4.0 | 0.0 | 9.7 | 3.1 |
| Arthropoda | Insecta | Diptera | Hybotidae |  | Hybotid Dance Fly spp. | 3.0 | 0.0 | 9.7 | 0.0 |
| Arthropoda | Insecta | Lepidoptera | Tortricidae | *Anopina ednana* | Leafroller Moth spp. | 3.0 | 4.2 | 0.0 | 6.2 |
| Arthropoda | Arachnida | Araneae | Araneidae | *Neoscona arabesca* | Arabesque Orbweaver | 3.0 | 4.2 | 3.2 | 3.1 |
| Arthropoda | Insecta | Coleoptera | Staphylinidae | *Eusphalerum pothos* | Rove Beetle spp. | 3.0 | 8.3 | 0.0 | 3.1 |
| Arthropoda | Insecta | Diptera | Empididae |  | Dance Fly spp. | 3.0 | 8.3 | 3.2 | 0 |
| Arthropoda | Insecta | Lepidoptera | Blastobasidae | *Pigritia laticapitella* | Moth spp. | 3.0 | 0.0 | 0.0 | 9.4 |
| Arthropoda | Arachnida | Araneae | Philodromidae | *Philodromus vulgaris* | Long-legged Crab Spider | 3.0 | 4.2 | 3.2 | 3.1 |
| Arthropoda | Insecta | Lepidoptera | Gracillariidae | *Caloptilia packardella* | Leaf Blotch Miner Moth spp. | 3.0 | 8.3 | 3.2 | 0.0 |
| Arthropoda | Insecta | Lepidoptera | Tortricidae | *Clepsis melaleucanus* | Black-patched Clepsis Moth | 3.0 | 8.3 | 3.2 | 0.0 |
| Arthropoda | Insecta | Lepidoptera | Crambidae | *Scoparia penumbralis* | Dark Brown Scoparia Moth | 3.0 | 0.0 | 6.5 | 3.1 |
| Arthropoda | Insecta | Coleoptera | Elateridae | *Athous acanthus* | Click Beetle spp. | 3.0 | 4.2 | 0.0 | 6.2 |
| Arthropoda | Insecta | Lepidoptera | Gelechiidae | *Coleotechnites piceaella* | Orange Spruce Needleminer | 3.0 | 12.5 | 0.0 | 0.0 |
| Arthropoda | Insecta | Hemiptera | Miridae | *Neolygus fagi* | Plant Bug spp. | 3.0 | 0.0 | 9.7 | 0.0 |
| Arthropoda | Insecta | Diptera | Chironomidae |  | Non-biting midges spp. | 3.0 | 8.3 | 0.0 | 3.1 |
| Arthropoda | Insecta | Hemiptera | Miridae | *Blepharidopterus provancheri* | Plant Bug spp. | 3.0 | 0.0 | 3.2 | 6.2 |
| Arthropoda | Insecta | Lepidoptera | Erebidae | *Zanclognatha laevigata* | Variable Fan-Foot | 3.0 | 0.0 | 9.7 | 0.0 |
| Arthropoda | Insecta | Lepidoptera | Gelechiidae | *Chionodes mediofuscella* | Black-smudged Chionodes Moth | 3.0 | 0.0 | 9.7 | 0.0 |
| Arthropoda | Arachnida | Araneae |  |  | Spider spp. | 3.0 | 4.2 | 3.2 | 3.1 |
| Arthropoda | Insecta | Hymenoptera | Braconidae |  | Braconid Wasp spp. | 3.0 | 8.3 | 3.2 | 0.0 |
| Arthropoda | Insecta | Diptera | Limoniidae |  | Limoniid Crane Fly spp. | 3.0 | 8.3 | 3.2 | 0.0 |
| Arthropoda | Arachnida | Araneae | Clubionidae | *Clubiona spiralis* | Leaf-curling Sac spider spp. | 3.0 | 4.2 | 3.2 | 3.1 |
| Arthropoda | Insecta | Lepidoptera | Tortricidae | *Olethreutes appendiceum* | Serviceberry Leafroller | 3.0 | 8.3 | 3.2 | 0.0 |
| Arthropoda | Insecta | Lepidoptera | Erebidae | *Lymantria dispar* | Spongy Moth | 3.0 | 12.5 | 0.0 | 0.0 |
| Arthropoda | Insecta | Diptera | Mycetophilidae | *Mycetophila fungorum* | Fungus Gnat spp. | 3.0 | 0.0 | 0.0 | 9.4 |
| Arthropoda | Insecta | Hymenoptera | Ichneumonidae |  | Ichneumonid Wasp spp. | 3.0 | 8.3 | 0.0 | 3.1 |
| Arthropoda | Insecta | Hymenoptera | Braconidae | *Diolcogaster sp.* | Braconid Wasp spp. | 3.0 | 0.0 | 6.5 | 3.1 |
| Arthropoda | Insecta | Lepidoptera | Gelechiidae |  | Twirler Moth spp. | 3.0 | 4.2 | 6.5 | 0.0 |
| Arthropoda | Insecta | Lepidoptera | Gelechiidae | *Dichomeris punctipennella* | Many-spotted Dichomeris Moth | 3.0 | 4.2 | 0.0 | 6.2 |
| Arthropoda | Insecta | Lepidoptera | Geometridae | *Operophtera bruceata* | Bruce Spanworm Moth | 3.0 | 12.5 | 0.0 | 0.0 |
| Arthropoda | Insecta | Hemiptera | Reduviidae | *Zelus luridus* | Pale Green Assasin Bug | 3.0 | 8.3 | 0.0 | 3.1 |
| Arthropoda | Insecta | Lepidoptera | Geometridae |  | Geometer Moth spp. | 2.0 | 8.3 | 0.0 | 0.0 |
| Arthropoda | Arachnida | Araneae | Linyphiidae | *Neriene radiata* | Filmy Dome Spider | 2.0 | 8.3 | 0.0 | 0.0 |
| Arthropoda | Insecta | Lepidoptera | Gelechiidae |  | Twirler Moth spp. | 2.0 | 8.3 | 0.0 | 0.0 |
| Arthropoda | Insecta | Lepidoptera | Gelechiidae | *Coleotechnites apicitripunctella* | Green Hemlock Needleminer | 2.0 | 8.3 | 0.0 | 0.0 |
| Arthropoda | Insecta | Diptera | Iteaphila.group | *Iteaphila nitidula* | Dance Fly spp. | 2.0 | 8.3 | 0.0 | 0.0 |
| Arthropoda | Insecta | Lepidoptera | Geometridae | *Melanolophia canadaria* | Canadian Melanolophia Moth | 2.0 | 4.2 | 0.0 | 3.1 |
| Arthropoda | Insecta | Diptera | Tachinidae |  | Bristle Fly spp. | 2.0 | 4.2 | 0.0 | 3.1 |
| Arthropoda | Insecta | Coleoptera | Coccinellidae | *Chilocorus stigma* | Twice-stabbed Lady Beetle | 2.0 | 4.2 | 0.0 | 3.1 |
| Arthropoda | Arachnida | Opiliones | Caddidae | *Caddo agilis* | Agile Harvestman | 2.0 | 0.0 | 0.0 | 6.2 |
| Arthropoda | Insecta | Diptera | Empididae |  | Dance Fly spp. | 2.0 | 4.2 | 3.2 | 0.0 |
| Arthropoda | Insecta | Diptera | Dolichopodidae |  | Long-legged Fly spp. | 2.0 | 0.0 | 6.5 | 0.0 |
| Arthropoda | Insecta | Lepidoptera | Gelechiidae | *Chionodes obscurusella* | Boxelder Leafworm Moth | 2.0 | 8.3 | 0.0 | 0.0 |
| Arthropoda | Insecta | Diptera | Bibionidae | *Dilophus femoratus* | March Fly spp. | 2.0 | 8.3 | 0.0 | 0.0 |
| Arthropoda | Arachnida | Araneae | Cybaeidae | *Cryphoeca montana* | Soft Spider spp. | 2.0 | 0.0 | 0.0 | 6.2 |
| Arthropoda | Insecta | Plecoptera | Nemouridae | *Ostrocerca complexa* | Notched Forestfly | 2.0 | 4.2 | 3.2 | 0.0 |
| Arthropoda | Insecta | Hymenoptera | Ichneumonidae |  | Ichneumonid Wasp spp. | 2.0 | 0.0 | 3.2 | 3.1 |
| Arthropoda | Arachnida | Araneae | Araneidae | *Araneus nordmanni* | Nordmann’s Orbweaver | 2.0 | 0.0 | 3.2 | 3.1 |
| Arthropoda | Insecta | Diptera | Sciaridae |  | Dark-winged Fungus Gnat spp. | 2.0 | 0.0 | 3.2 | 3.1 |
| Arthropoda | Arachnida | Araneae | Theridiidae | *Theridion glaucescens* | Cobweb spider spp. | 2.0 | 8.3 | 0.0 | 0.0 |
| Arthropoda | Insecta | Diptera | Simuliidae | *Simulium venustum* | White-stockinged Black Fly | 2.0 | 4.2 | 3.2 | 0.0 |
| Arthropoda | Arachnida | Araneae | Theridiidae | *Platnickina alabamensis* | Cobweb spider spp. | 2.0 | 8.3 | 0.0 | 0.0 |
| Arthropoda | Insecta | Coleoptera | Tenebrionidae | *Isomira quadristriata* | Comb-clawed Darkling Beetle spp. | 2.0 | 8.3 | 0.0 | 0.0 |
| Arthropoda | Insecta | Hymenoptera | Braconidae |  | Braconid Wasp spp. | 2.0 | 4.2 | 3.2 | 0.0 |
| Arthropoda | Insecta | Coleoptera | Elateridae |  | Click Beetle spp. | 2.0 | 8.3 | 0.0 | 0.0 |

| **TABLE S8.** PERMANOVA results indicate moderate dissimilarity in black-throated blue warbler diets between survey periods after excluding HY (hatch year) birds. All variables contained three levels (i.e., balanced design), and tests were conducted at the level of prey species. | | | | | | | |
| --- | --- | --- | --- | --- | --- | --- | --- |
|  | **PERMANOVA** | | |  | **Multivariate dispersion** | | |
| **Parameter** | **Pseudo-*F*** | **R^2^** | ***p*-value** | | | ***F*** | ***p*-value** |
| Survey period | 8.4 | 0.19 | 0.078 | | | 8.6 | < 0.001 |
| Elevation | 4.9 | 0.11 | 0.194 | | | 0.7 | 0.519 |
| Age group | 4.4 | 0.05 | 0.305 | | | 0.1 | 0.963 |
| Survey period * Elevation | -3.6 | -0.16 | 0.978 | | |  |  |

| **TABLE S9.** Frequency of occurrence (%) of all prey orders identified in the diets of black-throated blue warblers during the late survey period by age group (HY = hatch year [juveniles], AHY = after hatch year [adults] at the Hubbard Brook Experimental Forest, New Hampshire, USA. Total late is the percentage of the total number of samples in the late survey period across age groups that included a given order. | | | | |
| --- | --- | --- | --- | --- |
| **Order** | **Description** | **Total late** | **HY** | **AHY** |
| Lepidoptera | butterflies and moths | 32 | 100 | 100 |
| Araneae | spiders | 31 | 100 | 96.3 |
| Diptera | true flies | 29 | 100 | 88.9 |
| Hemiptera | true bugs | 16 | 40 | 51.9 |
| Hymenoptera | sawflies, wasps, bees, and ants | 15 | 40 | 48.1 |
| Psocodea | barklice, booklice, parasitic lice | 14 | 100 | 33.3 |
| Coleoptera | beetles | 10 | 0 | 37 |
| Plecoptera | stoneflies | 3 | 0 | 11.1 |
| Neuroptera | lacewings, mantidflies, antlions, and their relatives | 3 | 20 | 7.4 |
| Sarcoptiformes | mites | 2 | 0 | 7.4 |
| Entomobryomorpha | springtails | 2 | 0 | 7.4 |
| Trombidiformes | mites | 2 | 20 | 3.7 |
| Opiliones | harvestman spiders | 1 | 20 | 0 |
| Poduromorpha | springtails | 1 | 0 | 3.7 |

| **TABLE S10.** Rarefied estimates of species richness, Shannon diversity (exponentiated Shannon entropy), and Simpson diversity (inverse Simpson concentration) for prey species identified in the diets of black-throated blue warblers grouped into three survey periods. Estimates were rarefied to the minimum sample size (early period; *n* = 24) to facilitate comparisons between survey periods. Rarefied numbers give the mean estimate along with 95% confidence intervals. | | |
| --- | --- | --- |
| **Survey Period** | **Metric** | **Interpolated estimate (*n* = 24)** |
| Early | Species richness | 203 (188.9, 217.1) |
| Mid |  | 164.3 (148.6, 180.0) |
| Late |  | 164.8 (150.7, 179.0) |
| Early | Shannon diversity | 123.6 (112.7, 134.6) |
| Mid |  | 100.1 (90.0, 110.1) |
| Late |  | 106.1 (95.0, 117.3) |
| Early | Simpson diversity | 75 (66.2, 83.7) |
| Mid |  | 61.5 (56.0, 66.9) |
| Late |  | 67.3 (59.7, 74.9) |

**B. Supplementary Figures**


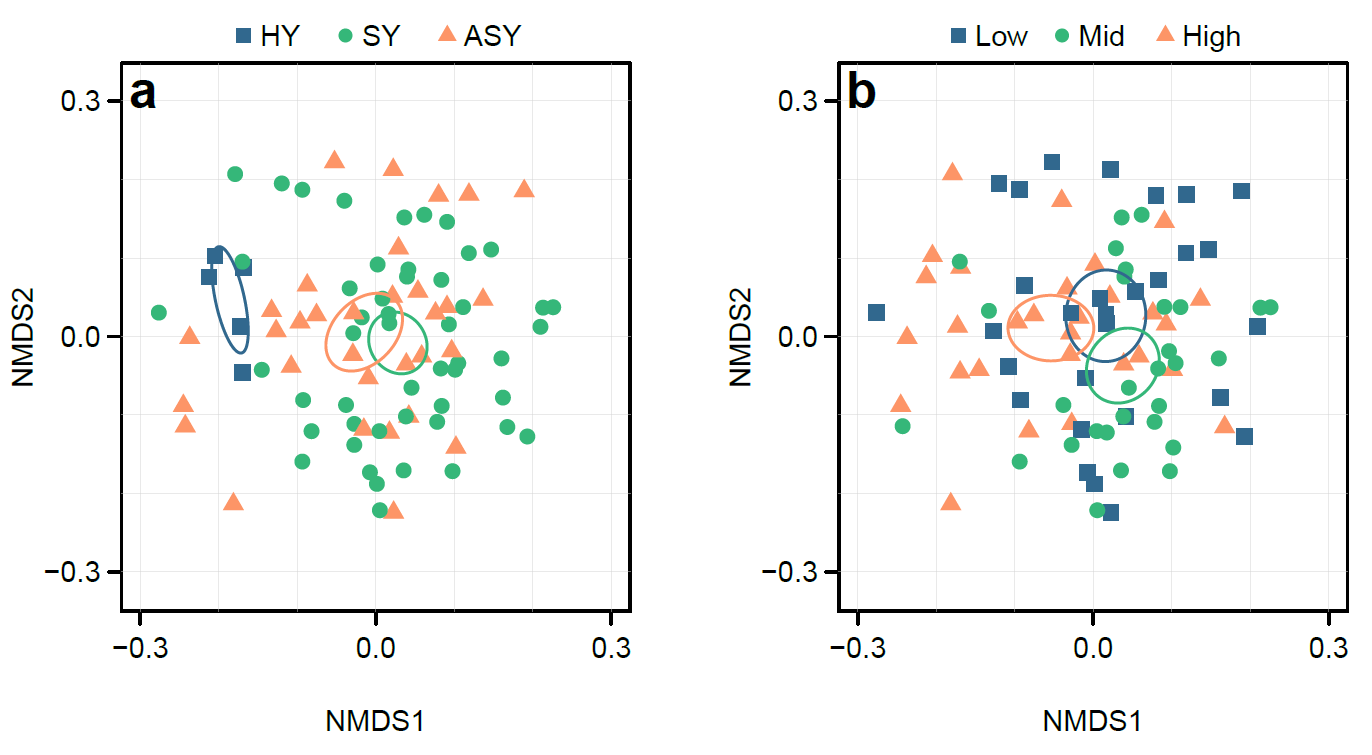


**Figure S1.** Non-metric multidimensional scaling (NMDS) plot of diet composition shows a) limited overlap between the diets of hatch year (HY) black-throated blue warblers relative to second year (SY) and after second year (ASY) adults and b) some overlap across elevation zones at the Hubbard Brook Experimental Forest, New Hampshire, USA. Points represent species-level diet composition within samples displayed with 95% confidence ellipses.


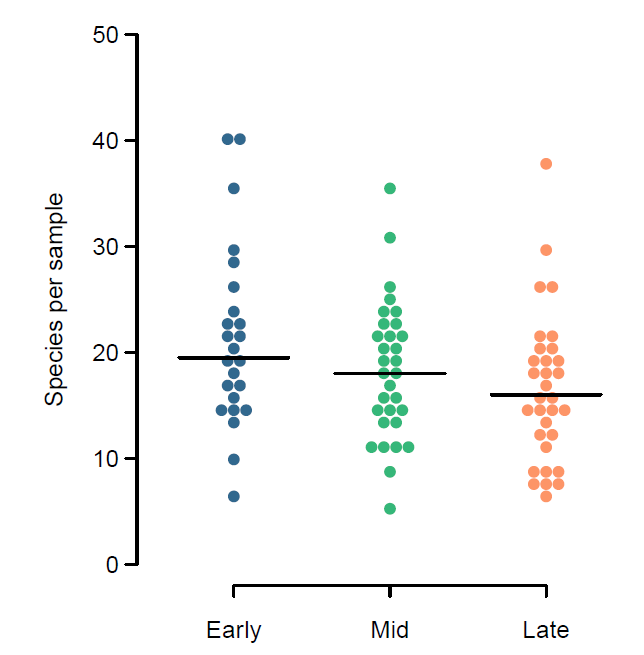


**Figure S2.** The number of prey species per sample showed limited differences between survey periods for black-throated blue warblers at the Hubbard Brook Experimental Forest, New Hampshire, USA. Points represent the number of species in each fecal sample, and lines represent the 50^th^ quartiles.


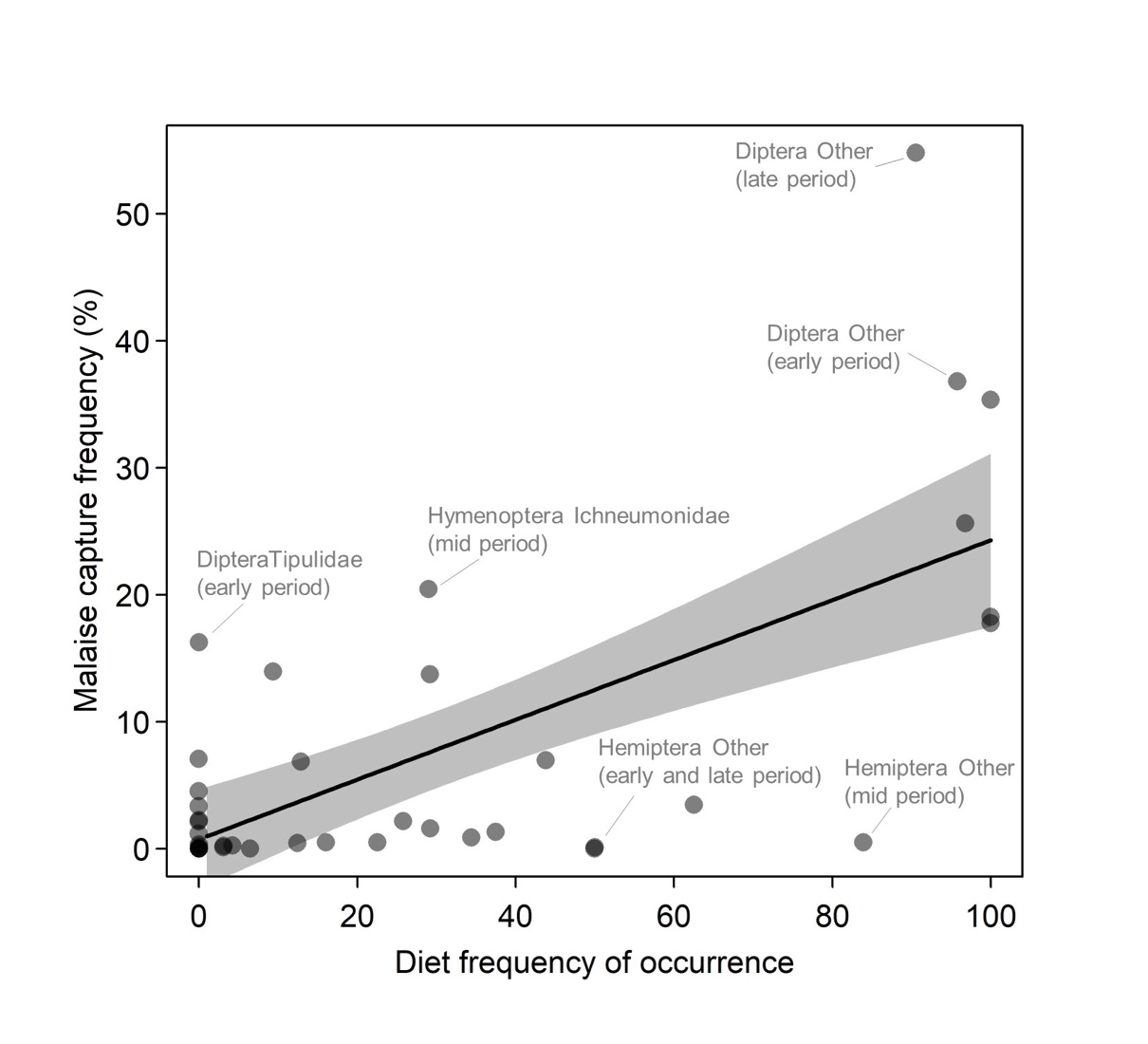


**Figure S3.** Comparison of the frequency of occurrence of prey items in the diets of black-throated blue warblers with capture frequency of focal arthropod families identified in Malaise traps (outliers labeled) at the Hubbard Brook Experimental Forest, New Hampshire, USA.
